# The Influence of Plant Extracts and Phytoconstituents on Antioxidant Enzymes Activity and Gene Expression in the Prevention and Treatment of Impaired Glucose Homeostasis and Diabetes Complications

**DOI:** 10.3390/antiox10030480

**Published:** 2021-03-18

**Authors:** Mirjana Mihailović, Svetlana Dinić, Jelena Arambašić Jovanović, Aleksandra Uskoković, Nevena Grdović, Melita Vidaković

**Affiliations:** Department of Molecular Biology, Institute for Biological Research “Siniša Stanković”—National Institute of Republic of Serbia, University of Belgrade, Bulevar Despota Stefana 142, 11060 Belgrade, Serbia; mista@ibiss.bg.ac.rs (M.M.); sdinic@ibiss.bg.ac.rs (S.D.); jelena.arambasic@ibiss.bg.ac.rs (J.A.J.); auskokovic@ibiss.bg.ac.rs (A.U.); nevenag@ibiss.bg.ac.rs (N.G.)

**Keywords:** plant extracts, phytoconstituents, diabetes, diabetic complications, oxidative stress, antioxidant enzymes, nutrigenomics

## Abstract

Diabetes is a complex metabolic disorder resulting either from insulin resistance or an impaired insulin secretion. Prolonged elevated blood glucose concentration, the key clinical sign of diabetes, initiates an enhancement of reactive oxygen species derived from glucose autoxidation and glycosylation of proteins. Consequently, chronic oxidative stress overwhelms cellular endogenous antioxidant defenses and leads to the acute and long-standing structural and functional changes of macromolecules resulting in impaired cellular functioning, cell death and organ dysfunction. The oxidative stress provoked chain of pathological events over time cause diabetic complications such as nephropathy, peripheral neuropathy, cardiomyopathy, retinopathy, hypertension, and liver disease. Under diabetic conditions, accompanying genome/epigenome and metabolite markers alterations may also affect glucose homeostasis, pancreatic β-cells, muscle, liver, and adipose tissue. By providing deeper genetic/epigenetic insight of direct or indirect dietary effects, nutrigenomics offers a promising opportunity to improve the quality of life of diabetic patients. Natural plant extracts, or their naturally occurring compounds, were shown to be very proficient in the prevention and treatment of different pathologies associated with oxidative stress including diabetes and its complications. Considering that food intake is one of the crucial components in diabetes’ prevalence, progression and complications, this review summarizes the effect of the major plant secondary metabolite and phytoconstituents on the antioxidant enzymes activity and gene expression under diabetic conditions.

## 1. Introduction

**Figure 1:**
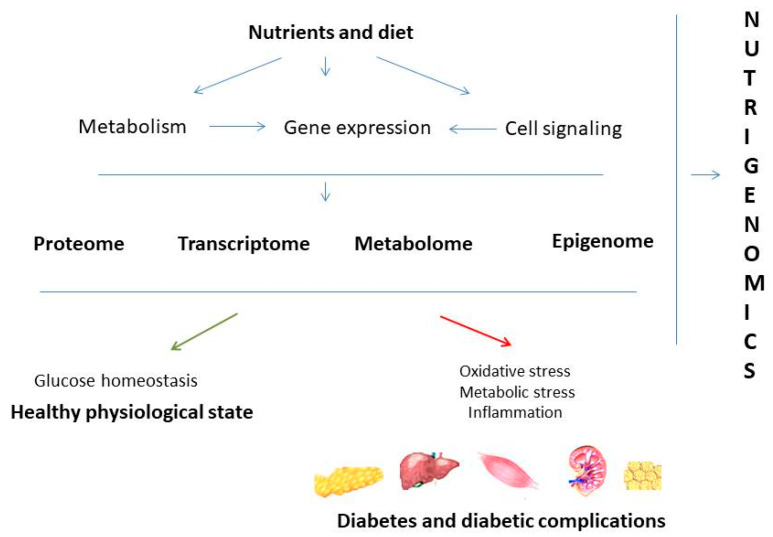
Concept of nutrigenomics in diabetes. Nutrients present in food can affect gene expression, cell signaling pathways, and cell metabolism. Those nutrient-gene interactions reflect on epigenome, transcriptome, proteome, and metabolome. Those interactions can be protective and lead to the healthy physiological state or can increase inflammation, oxidation, and metabolic stress which further lead to disturbed glucose homeostasis and progression to diabetes and diabetic complications.

Environmental influences and nutritional intake are very much responsible for development of different diseases as two main factors that might affect health of an individual. To solve a complex interplay between environmental influences, nutrition and human health, the scientific field of nutrigenomics has started to develop. The last decade of nutrigenomics development provided evidence of the impact of nutrients and bioactive food compounds on gene expression and gene regulation, protein expression, epigenetic alterations, and metabolite changes. By understanding the interaction between genome, proteome, and metabolome with nutrients and bioactive food components, nutrigenomics connects nutrition and human health in order to classify dietary compounds with beneficial or injury effects [1]. In line with that, it was shown that diet is an important component that influences the onset of diabetes and development of its complications (Figure 1).

Diabetes is a metabolic disorder caused by impaired insulin secretion or insulin action bringing the improper balance of glucose homeostasis. According to the International Diabetes Federation report, 451 million adult people worldwide are diagnosed as diabetic, with a prediction of even 693 million cases by 2045 [2] indicating the epidemic scale of the disease. Hyperglycemia in type 1 diabetes (T1D), resulting from the autoimmune destruction of pancreatic β-cells, is controlled by direct insulin administration, but this therapy cannot accomplish exact physiological control of blood glucose concentrations and complications continue to progress [3]. On the other hand, blood glucose levels in type 2 diabetes (T2D) are regulated by oral antihyperglycemic therapy [4]. Even though development of T2D has been shown to be delayed by oral antihyperglycemic treatment, β-cell function continues to decline in diabetic patients with progressive failure of insulin secretion [5]. To overcome limitations in current diabetes therapy, medicinal plants could be potentially used as auxiliary medicines with additive effects on antidiabetic drugs action. Although not equally effective in lowering glucose level in comparison to insulin and hypoglycemic drugs, medicinal plants exhibit various effects such as antioxidant and cholesterol-lowering effects, others have been shown to contribute to reduction of insulin resistance and β-cell regeneration, while some have been shown to contribute to symptomatic relief and prevention of diabetes complications [6,7].

Several compounds or natural products have been reported as analogs of synthetic antidiabetic drugs with advantage of having few or no side effects [8]. Nutrients, natural products and phytoconstituents can influence gene expression and gene regulation that contribute to diabetes risk, progression and complications thus affecting altered glucose homeostasis, pancreatic β-cells, liver, muscle, hypothalamus, and adipose tissue [1]. Consequently, it becomes evident that dietary intervention with bioactive food compound could have a great impact and beneficial effects in diabetic condition. Therefore, the main focus of the present review is related to antidiabetic and antioxidant properties of plant extracts and phytoconstituents and their influence on antioxidant enzymes activity and gene expression in the prevention and treatment of diabetes and its complications. The retrospective of the great work that has been promoted in the last decade regarding nutritional influence on diabetes pathology is essential, since it can boost the use of dietetic scheme for treatment and attenuation of diabetes and its complication making a critical step in upgrading the quality of life for diabetic patients. Furthermore, some of defined bioactive components isolated from different plants might be used as a solid basis for synthetizing novel antidiabetic drugs with a final aim of improving diabetic condition and diminishing any drug-induced side effects.

## 2. Diabetes and Oxidative Stress

Glucose homeostasis is regulated by several factors and mechanisms and any alteration of those factors leads to impaired glucose homeostasis and consequently to diabetes, obesity, and other disorders [9]. Prolonged hyperglycemia and hyperlipidemia are associated with oxidative stress which is presently seen as an important piece of the puzzle for understanding the origin, development, and pathogenesis of T1D and T2D and other adverse effects including diabetic complications such as nephropathy, neuropathy, cardiovascular and liver diseases. Moreover, sustained oxidative stress triggers the expression of different inflammation-regulated genes and activates pathogenic proinflammatory pathways that also contribute to a variety of diabetic complications [10]. Reactive oxidative (ROS) and reactive nitrogen species (RNS) are reactive, short lived and very unstable chemical entities that are produced as a result of normal physiological processes in cells and play important roles in cellular signaling, gene transcription and the immune response [11]. Excessive production or accumulation of reactive species induces cell damage by oxidation of cell components and molecules. Both external and internal stimuli can produce free radicals and imbalance between overproduction and/or insufficient removal of highly reactive species (ROS and RNS) results in oxidative stress.

**Figure 2:**
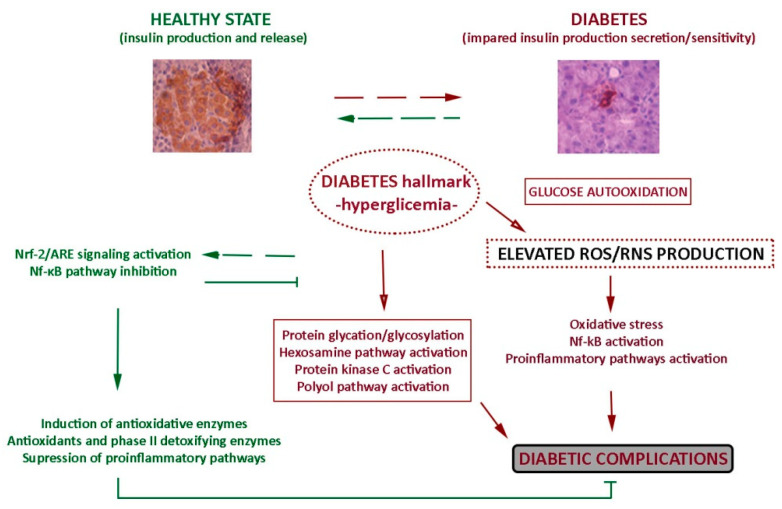
Hyperglycemia, oxidative stress, and diabetic complications. Hyperglycaemia and glucose autooxidation are the main sources of free radicals in diabetic condition. Hyperglycaemia contributes to pathogenesis of diabetes, not only by free radical generation, but also through protein glycation and glycosylation, augmented metabolism of glucose through the hexosamine pathway, increased activation of the polyol pathway by unused glucose, glucose-mediated activation of protein kinase C. Under normal circumstances the concentration of ROS is modulated by antioxidant enzymes, antioxidants, and phase II detoxifying enzymes. Those cellular antioxidant defense systems are regulated by a master regulator Nrf-2, as well as by NF-κB. Nrf-2 (nuclear factor erythroid 2-related factor 2), NF-κB (nuclear factor kappa B), ARE (antioxidant response element). Immunohistological images of insulin (brown) present in pancreatic islets are taken from Mihailović et al., “Protective Effects of the Mushroom *Lactarius deterrimus* Extract on Systemic Oxidative Stress and Pancreatic Islets in Streptozotocin-Induced Diabetic Rats”, Journal of Diabetes Research, ID 576726, 10 pages, 2015. https://doi.org/10.1155/2015/576726 (accessed on 5 March 2021).

Although different mechanisms may contribute to the formation of these reactive species, glucose autooxidation is supposed to be the main source of free radicals in diabetic condition. Catalyzed by trace amounts of transition metals, glucose oxidation generates superoxide anion radicals and protein reactive ketoaldehydes. Dismutation of superoxide anion radicals into hydrogen peroxide (H_2_O_2_) leads to the further production of particularly reactive hydroxyl radicals [12]. Also, superoxide anion radicals can react with nitric oxide to form peroxynitrite radicals [12]. Under normal circumstances, endogenous antioxidant enzymes and nonenzymatic antioxidants remove and protect the cells from the damaging effects of free radicals. The concentration of ROS is modulated by antioxidant enzymes such as catalase (CAT), glutathione peroxidase (GPx), glutathione reductase (GR), superoxide dismutase (SOD), and by nonenzymatic antioxidants such as reduced glutathion (GSH) [13]. Those enzymes play a key role in cellular homeostasis and perform together in the metabolic pathway of free radicals. Cytosolic SOD (SOD-1 or CuZnSOD) and mitochondrial SOD (SOD-2 or MnSOD) convert intracellular superoxide radicals into H_2_O_2_ which can be detoxified into H_2_O by GPx and CAT. Besides, activation of phase II detoxifying enzymes such as glutathione S-transferases (GSTs), heme oxygenase-1 (HO-1) and nicotinamide adenine dinucleotide phosphate (NAD(P)H) quinone oxidoreductase 1 (NQO-1) is an important cellular defense system against oxidative and electrophilic insults [14]. Those cellular antioxidant defense systems are regulated by a master regulator known as nuclear factor erythroid 2-related factor 2 (Nrf-2), as well as by the nuclear factor kappa B (NF-κB) which also orchestrates a wide range of inflammatory responses by regulating pro-inflammatory cytokine gene expression and adhesion molecules [15,16,17]. In untreated diabetes, the protein levels and enzymatic activities of antioxidant enzymes are decreased and consequently an imbalance in production and removal of reactive species results in cell dysfunction and destruction and finally in tissue injury [18]. High glucose level contributes to pathogenesis of diabetes, not only by free radical generation, but also through non-enzymatic protein glycation [19], augmented metabolism of glucose through the hexosamine pathway [20], increased activation of the polyol pathway by unused glucose, glucose-mediated activation of protein kinase C [21] and through increased lipid peroxidation, resulting in damaging of enzymes, the β-cell dysfunction, impaired glucose tolerance, increased insulin resistance, increased inflammation and vascular dysfunction (Figure 2).

In addition to endogenous mechanisms responsible for the removal of increased ROS/RNS levels and normalization of distracted redox balance, in the last decade more attention has been dedicated to the pharmacology of antioxidants. Herein, diet provided mainly by medicinal herbs and phytoconstituents can be a balancing source of exogenous antioxidants and use of medicinal plants has the advantage of with few or no side effects [22]. Given that hyperglycaemia-induced oxidative stress affects majority of tissues and organs in diabetic patients, use of natural products with antidiabetic and antioxidant properties could have a multiple beneficial effects. Numerous in vitro and in vivo studies demonstrated that plant extracts and their constituents (such as polyphenols and its derivatives, flavonoids, carbohydrates, glycosides, alkaloids, saponins, peptidoglycans, minerals, and vitamins) protect cells from oxidative damage directly through free radical scavenging activities or indirectly by modulating signal transduction pathways and expression of redox-related genes [22,23]. To gain a closer insight into the effects of nutrients on the gene expression in diabetes pathogenesis, this review is particularly focused on the effects of antidiabetic plants and derived phytoconstituents on the expression and the activity of antioxidant enzymes. Additionally, dietary-induced epigenetic changes may influence antioxidant enzymes’ gene expression, which is also discussed.

## 3. Plants with Antidiabetic and Antioxidant Properties Modulate Redox-Related Gene Expression

Studies of antidiabetic and antioxidant properties of plants traditionally used in alleviating diabetic symptoms revealed their potential to ameliorate oxidative stress-induced dysfunction of β- and endothelial cells, decrease of insulin secretion and low-density lipoprotein (LDL) augmentation [22,23]. To identify bioactive compounds with potential therapeutic significance, majority of those studies included phytochemical characterization of plant extracts as well. Medicinal plants and their mechanisms of antidiabetic action are listed below in Table 1, Table 2 and Table 3.

### 3.1. Plants with Protective Effects on Pancreas in Diabetic Condition

*Centaurium erythraea* Rafn (Common or Europian centaury) is traditionally used in Mediterranean countries for treating various ailments including diabetes which has stimulated efforts for studying the mechanisms of its antidiabetic action. Administration of *C. erythraea* aqueous extract (200 mg/kg/day, intraperitoneally (i.p)) for 30 days to streptozotocin (STZ)-induced diabetic rats significantly reduced blood glucose and malondialdehyde (MDA) levels, increased the activities of both enzymatic (SOD, CAT, GPx) and non-enzymatic (GSH) antioxidants in pancreas and minimized degenerative changes of pancreatic β-cells to near normal morphology [24]. Those results clearly indicated the therapeutic potential of *C. erythrea* in treating diabetes owing to decreased oxidative damage of pancreatic β-cells. In another study, application of *C. erythraea* extract (CEE) (100 mg/kg) for four weeks improved the levels of insulin, blood glucose, glycated hemoglobin, and lipid profile in serum of STZ-induced diabetic rats [25]. STZ-induced disruption of Langerhans islet cell content and morphology in rats was improved by CEE and was associated with its protective effect on the levels of insulin, p-Akt and GLUT-2 in diabetic islets [26]. At the same time, disturbance in CAT, MnSOD, and CuZnSOD enzyme activities were improved after CEE application. Furthermore, the CEE treatment improved the observed changes of the mRNA expression for CAT, MnSOD, CuZnSOD, GPx and GR enzymes in rat pancreatic β-cells. This study indicated that CEE improved the structural and functional properties of pancreatic β-cells, at least in part, by correcting the activities of redox-sensitive nuclear factor kappa B protein subunit 65 (NF-κB-p65), forkhead box O3 (FOXO3A), specificity protein 1 (Sp1), and Nrf-2 transcription factors and by modulating the activities of pro-survival protein kinase B (Akt), mitogen-activated protein kinase (p38) and extracellular signal-regulated kinase (ERK) kinases and islet-enriched pancreatic and duodenal homeobox 1 (Pdx-1) and musculoaponeurotic fibrosarcoma oncogene homolog A (MafA) regulatory factors. After pancreatic β-cell exposure to oxidative/nitrosative stress, CEE also improved insulin expression and secretion by decreasing DNA damage, protein S-glutathionylation, lipid peroxidation, by restoring GSH homeostasis in H_2_O_2_-treated β-cells and attenuating the sodium nitroprusside (SNP)-induced disturbance of the reduced/oxidized glutathione (GSH/GSSG) ratio in Rin-5F cells [27]. Additionally, CEE adjusted H_2_O_2_- and SNP-induced disturbance in mRNA and protein levels of GPx, CAT, MnSOD and CuZnSOD. In the same manner, CEE treatment also improved activities of the above-mentioned antioxidant enzymes. Based on phytochemical characterization, described protective effects of *C. erythraea* could be attributed to its predominant compounds such as secoiridoids (swertiamarin, gentiopicrin, sweroside, loganin, and secologanin) and polyphenolic compounds (p-coumaric, sinapic acid, caffeic acid, and ferulic), flavonoids (luteolin and apigenin), flavonols (rutin, quercetin, kaempferol, isoquercitrin, and astragalin), and flavanones (naringenin), as well as xanthones (desmethyleustomin, eustomin, decussatin and methylbellidifolin) [25].

*E. punctata, E. subcoriacea,* and *Eysenhardtia platycarpa* (Fabaceae), used in Mexico for the treatment of diabetes complications, were analyzed for the antioxidant activities in experimental model of diabetes [28]. Methanolic extracts of those plants protected rat pancreatic homogenate from 2,2-azo-bis(2-amidinopropane)dihydrochloride (AAPH)-induced damage. Two compounds (3-O-Acetyl-11alpha,12alpha-epoxy-oleanan-28,13beta-olide and (+)-catechin) isolated from the branches of *E. platycarpa* significantly increased GSH concentration alone and in co-treatment with AAPH in the pancreas homogenate model. Treatment of STZ-induced diabetic rats with 3-O-Acetyl-11alpha,12alpha-epoxy-oleanan-28,13beta-olide compound (100 mg/kg body weight, i.p. for 5 days) prevented hyperglycemia and significantly reduced level of thiobarbituric acid reactive substances (TBARS) and increased the activities of GPx and CAT in rats’ pancreas. *Castanea sativa* Mill. (Fagaceae) known as sweet chestnut, mainly grows in Mediterranean region of Europe and in some parts of Asia. Ethanolic extracts prepared from catkins, leaves and spiny burs of *C. sativa* displayed high in vitro free radical-scavenging activity correlated with their high phenolic and flavonoid content and prevented oxidative stress mediated β-cell death [29]. The improved viability of STZ-treated Rin-5F cells by chestnut extracts partially resulted from preservation of GSH content and reduction of DNA damage and lipid peroxidation. In addition, ethanolic extract from chestnut spiny burs improved insulin protein level and reduced activity of SODs and CAT, glutathione oxidation, NO-output and NF-kB-p65 activity in STZ-treated Rin-5F cells [30]. Together with chelating effects, high reducing power and radical scavenging activity, those findings suggest that chestnut extract probably acts through ROS scavenging activity, which correlates with its high phenolic (ellagic and galic acids and their derivatives) and flavonoid content [30]. *Solanum torvum* Swartz. Fruit, containing high levels of phenolic compounds mainly gallic acid, caffeic acid, rutinand catechin, has a great antidiabetic and antioxidant potential [31]. Methanol extract of *S. torvum* (STMe) was administered orally at two different doses (200 and 400 mg/kg/day) to STZ-induced diabetic rats for 30 days. Results revealed that STMe in both concentrations improved activities of CAT, SOD, and GPx in diabetic livers. Histological analysis of liver and pancreas strongly indicated cytoprotective action of STMe together with apparent β-cells regeneration after immunohistochemical staining of islets in STMe-treated diabetic rats (summarized in Table 1).

**Table 1:** Plants with protective effects on pancreas in diabetes pathogenesis.

Plant Extract	Model	Mechanism of Action	Reference
*Centaurium erythraea* Rafn Aqueous extract	STZ-induced diabetic rats	Reduces blood glucose level; reduces MDA level; induces GSH level and SOD, CAT, GPx activities; reduces degenerative changes of pancreatic islets.	[24]
Methanolic extract	STZ-induced diabetic rats	Improves islet cell content and insulin, GLUT-2, p-Akt levels.	[26]
Methanolic extract	STZ-treated Rin-5F cells	Increases cell viability, insulin secretion and mRNA level; reduces DNA damage, TBARS, GSSP, CAT and SOD activities; reduces mRNA of CAT, GPx, Mn/CuZnSOD; reduces NFκB-p65 and Nrf-2; induces Akt, ERK, p38, Pdx-1, MafA.	[26]
Methanolic extract	H_2_O_2_/SNP-treated Rin-5F cells	Reduces TBARS, GSSP; increases GSH; modulates activities of CAT, GPx, GR, Mn/CuZnSOD; down-regulates mRNA levels of Mn/CuZnSOD, GPx, CAT.	[27]
*Eysenhardtia platycarpa,* *Eysenhardtia punctate, Eysenhardtia subcoriacea* Methanolic extracts	Rat pancreas homogenate	Protection against (AAPH)-induced pancreas damage.	[28]
*Castanea sativa* Mill. Ethanolic extract	STZ-treated Rin-5F cells	Increases cell viability and insulin protein level; preserves GSH; reduces TBARS, GSSP, DNA damage.	[29,30]
*Solanum torvum* Swartz Methanolic extract	STZ-induced diabetic rats	Cytoprotection.	[31]

Abbreviations: STZ, streptozotocin; SNP, sodium nitroprusside; AAPH, 2,2-azo-bis(2-amidinopropane)dihydrochloride; SOD, superoxide dismutase; CAT, catalase; GPx, glutathione peroxidase; GR, glutathione reductase; GST, glutathione S-transferase; GSSP, glutathione disulfide; MDA, malondialdehyde; TBARS, thiobarbituric acid reactive substances; GSH, glutathione; NF-kB, nuclear factor kappa B; Nrf-2, nuclear factor erythroid 2-related factor 2; GLUT-2, glucose transporter 2; p-Akt, phospho-protein kinase B; ERK, extracellular signal-regulated kinase; p38, mitogen-activated protein kinase; Pdx-1, pancreatic and duodenal homeobox 1; MafA, musculoaponeurotic fibrosarcoma oncogene homolog A; H_2_O_2_, hydrogen peroxide; Rin-5F, rat pancreatic cell line.

### 3.2. Plants in the Service of Alleviation of Diabetic Complications

In addition to described antioxidant effects directed toward preserving the functioning of pancreatic β-cells, herbal extracts also contribute to alleviation of diabetic complications (summarized in Table 2). *C. erythraea* extract (CEE) applied in dose 100 mg/kg for 4 weeks to STZ-diabetic rats reduced oxidative stress biomarkers and protected red blood cell (RBC) proteins from damage by reducing non-enzymatic glycation and enzymatically regulated beta-N-acetylglucosamine (O-GlcNAc) glycosylation of proteins [25]. By increasing the activity of pro-survival Akt kinase in RBC and by reducing the level of serum α2-macroglobulin in diabetic animals, CEE exerted a potential to improve microcirculation and to provide an adequate oxygen supply in diabetic condition [25]. In vivo studies suggest that chestnut extract might possibly slow down the processes that cause diabetes onset and progression. Daily administration of *C. sativa* spiny burs ethanolic extract (60 mg/kg/i.p.) for 4 weeks to STZ-induced diabetic rats improved hyperglycemia and hyperlipidemia, as well as redox status in liver and kidney by lowering DNA damage, lipid peroxidation, GSH oxidation and by inducing Mn/CuZnSOD activity. Moreover, extract reduced protein glycation and O-glycosylation in both liver and kidney of diabetic rats accompanied by inhibition of receptor for advanced glycation end-product (RAGE)/NF-κB pathway [32]. In another study, *C. sativa* spiny burs ethanolic extract (60 mg/kg/i.p.) applied for 4 weeks to STZ-induced diabetic rats, alleviated liver damage by reducing collagen fiber deposition, O-glycosylation of SOD, CAT and NF-kB and by activating prosurvival Akt kinase [33]. Treatment with *C. sativa* extract lowered hepatic oxidative stress by decreasing glutathione disulfide (GSSP) and sulfhydryl group (-SH) content and by recovering mRNA levels and the activities of Mn/CuZnSOD and CAT [33].

Leave extract from *Cyclocarya paliurus*, common ingredient in functional foods in China, displayed high in vitro and in vivo antioxidant activities. Though phenolic compounds quercetin-3-O-glucuronide and quercetin-3-O-rhamnoside appeared to have a higher input in in vitro antioxidant activities of *C. paliurus*, correlation analysis revealed that another two phenolic compounds (kaempferol-3-O-glucoside and kaempferol-3-O-rhamnoside) from *C. paliurus* were responsible for the detected increase of blood SOD and GPx activities and decrease of MDA levels in C57BL/6 diabetic mice [34]). Medicinal plant *Lannea coromandelica* (Houtt.) Merr. is used for treating diabetes and heart disease in certain tribal communities of Bangladesh. Study of the antioxidant activities of *L. coromandelica* bark methanolic extract (LCBE) revealed its stimulating effect on the mRNA and protein levels of CAT, SOD1 and GPx1, as well as of phase II detoxifying enzymes (HO-1), through up-regulation of the Nrf-2-mediated pathway in monocyte/macrophage-like RAW 264.7 cells [35]. The ability of LCBE to protect cells from oxidative stress could be attributed to (−)-epigallocatechin-3-gallate (EGCG), gallic acid, catechin, chlorogenic and caffeic acids identified in LCBE by HPLC analysis. Administration of aqueous extract *Pueraria tuberosa* (50 mg/100 g or 100 mg/100 g, orally) for 20 days to STZ-diabetic rats significantly elevated the activity of CAT, SOD and GPx antioxidant enzymes, suppressed total ROS generation and the level of lipid peroxides in kidney tissue suggesting nephroprotective potential of *P. tuberosa* by virtue of different bioactive compounds with antioxidant activity [36]. Namely gas chromatography-mass spectrometry (GC-MS) analysis of *P. tuberosa* extract revealed the presence of 37 compounds where 2,3-dihydro-3,5-dihydroxy-6-methyl-4H-pyran-4-one, 5-Hydroxymethylfurfural, n-Hexadecanoic acid and 9-Octadecenoic acid (Z) were the most abundant. *Gongronema latifolium* Benth. leaves are traditionally used for diabetes treatment in Nigeria and West Africa. Study performed to evaluate its antidiabetic and antioxidant effects revealed that administration of *G. latifolium* leaf aqueous extract improved activity of antioxidant enzymes (SOD, GPx and CAT) as well as restored the changes in the levels of fasting blood glucose (FBG) in kidneys of alloxan-induced diabetic rats [37].

Glucose lowering, hypolipidemic, and antioxidant effects were reported for the *Vitellaria paradoxa*, a medicinal plant used in Africa for diabetes management. Treatment of alloxan-induced diabetic rats for 14 days with aqueous extract of *V. paradoxa* bark (125, 250 and 500 mg/kg bw, orally) increased the level of GSH and the activities of SOD and CAT enzymes in the liver of diabetic rats, suggesting that *V. paradoxa* extract may reduce diabetes-associated complications [38]. By increasing GST activity in H_2_O_2_-treated HepG2 cells, Terminalia chebula fruit ethyl acetate extract also displayed hepatoprotective potential [39]. A fraction from *Annona crassiflora* Mart. fruit peel (Ac-Pef) enriched in polyphenols, represents another promising natural source of bioactive components such as (epi)catechin, procyanidin B2, chlorogenic acid, and caffeoyl-glucoside with hepatoprotective effects. Treatment of STZ-diabetic rats for 30 days with Ac-Pef ethanolic extract, stimulated activities and expression of SOD, CAT, and GPx in the liver [40]. Aqueous extract of *Scoparia dulcis* was effective in attenuating hyperglycemia and protecting diabetic rats from deleterious effects of ROS [41]. Administration of *S. dulcis* extract (200 mg/kg, daily for six weeks) to STZ-diabetic rats significantly reduced blood glucose, TBARS, glycosylated hemoglobin, sorbitol dehydrogenase, hydroperoxides, and significantly increased the level of insulin in plasma and activities of GSH, GST, and GPx in the liver. *Alhagi maurorum* (camel thorn plant) is widely distributed plant in Europe, Asia, Africa, and the Middle East, and with a potential to alleviate diabetes-associated complications due to hypoglyceimic, hypolipidemic and antioxidant effects detected in STZ-diabetic rats [42]. The same study showed that both aqueous and ethanolic extracts of *A. maurorum* reduce oxidative stress in the liver by upraising the level of GSH, decreasing the MDA level and by inducing the activities of GPx and GST antioxidant enzymes that were inhibited in diabetic rats. The authors suggest that the antioxidant effect of *A. maurorum* extract could arise from gallic acid, rutin, and quercetin detected in both extracts [42].

*Ixeris gracilis* DC. Stebbins (Asteraceae), traditionally used by local communities of Meghalaya in India, is listed as plant with antidiabetic potential. Administration of methanolic extract (250 mg/kg) for 12 days to alloxan-induced diabetic mice improved glucose tolerance, glycemic control, and activities of GPx and SOD in the liver, kidney, and brain [43]. *Trigonella foenum graecum* L. (fenugreek) is being used as folk medicine for diabetes treatment and several studies have demonstrated that fenugreek seed extract, mucilage of seeds and leaves have hypoglycemic effect in experimental diabetic animals and in humans [44,45]. A study by Sharma and coworkers [46] showed that beside hypoglycemic effect, *T. foenum graecum* aqueous extract increases activities and mRNA levels of SOD, GPx and CAT in the liver and the brain of alloxan-induced diabetic rats. In another study, *T. foenum graecum* aqueous seed extracts lowered blood glucose levels, total cholesterol, triglycerides, and increased high density lipoprotein (HDL) cholesterol in STZ-diabetic rats. The hypoglycemic effect of *T. foenum graecum* probably resulted from its bioactive constituents such as 4 hydroxy isoleucine and steroid saponin trigonelline [47].

**Table 2:** Protective effects of plant extracts on liver, kidney, and circulation in diabetic condition.

Plant Extract	Target	Model	Mechanism of Action	Reference
*Castanea sativa* Mill. Ethanolic extract	Liver/Kidney	STZ- diabetic rats	Improves hyperglycemia and hyperlipidemia; reduces DNA damage and GSSP; improves Mn/CuZnSOD activities; inhibits RAGE/NF-κB pathway.	[32]
Ethanolic extract	Liver	STZ- diabetic rats	Induces mRNA levels and the activities of Mn/CuZnSOD and CAT; reduces SOD, CAT and NF-kB glycosilation; increases p-Akt level.	[33]
*Solanum torvum* Swartz Methanolic extract	Liver	STZ- diabetic rats	Improves activities of SOD, CAT, GPx; cytoprotection.	[31]
*Gongronema latifolium* Benth. Aqueous extract	Kidney	Alloxan-induced diabetic rats	Restores the alterations in FBG and activities of CAT, SOD, GPx.	[37]
*Pueraria tuberosa* Aqueous extract	Kidney	STZ-induced diabetic rats	Increases activity of SOD, CAT, GPx; suppresses total ROS generation and lipid peroxides.	[36]
*Vitellaria paradoxa* Aqueous extract	Liver	Alloxan-induced diabetic rats	Increases GSH and activities of CAT and SOD.	[38]
*Terminalia chebula* Ethyl acetate extract	Liver	H_2_O_2_-treated HepG2 cells	Increases GST activity.	[39]
*Annona crassiflora* Mart. Ethanolic extract	Liver	STZ-diabetic rats	Stimulates GPx, SOD, CAT activity and expression.	[40]
*Scoparia dulcis* Aqueous extract	Liver	STZ-diabetic rats	Reduces blood glucose, glycosylated hemoglobin, TBARS, hydroperoxides and sorbitol dehydrogenase; increases insulin in plasma and activities of GPx, GST and GSH in the liver.	[41]
*Alhagi maurorum* Aqueous/ethanolic extracts	Liver	STZ- diabetic rats	Increases GSH; decreases MDA; induces GPx and GST activities.	[42]
*Trigonella foenum graecum* Aqueous extract	Liver	Alloxan- diabetic rats	Hypoglycemic effect; increases activities and mRNA levels of SOD, GPx and CAT.	[46]
Aqueous extract	Circulation	STZ-diabetic rats	Loweres blood glucose levels, total cholesterol, triglycerides and increases HDL.	[47]
*Ixeris gracilis DC. Stebbins* Methanolic extract	Liver/Kidney	Alloxan-diabetic rats	Improves glucose tolerance and glycemic control and the activities of GPx and SOD.	[43]
*β-glucan-enriched extract* (1,3/1,4 β-glucan and a small amount of 1,6-linked glucose residues)	Liver/ Kidney Liver	STZ- diabetic rats STZ- diabetic rats	Improves hyperglycemia and hyperlipidemia; reduces DNA damage and TBARS; induces MnSOD, CuZnSOD and CAT activities; reduces glycation of serum proteins and reduces glycosylation of MnSOD, CuZnSOD, CAT. Attenuates inflammatory response by normalizing acute-phase proteins (α2-M and albumin) in serum; induces mRNA of anti-inflammatory cytokines (IL-10 and IL-4) and inhibites RAGE/NF-kB signaling in liver.	[48] [49] [10]
*Lannea coromandelica* Houtt. Methanolic extract	Mon./ Macro.-like cells	APPH-treated RAW 264.7 cells	Induces mRNA and protein levels of SOD1, CAT, GPx1 and HO-1; enhances Nrf-2 pathway.	[35]
*Centaurium erythraea* Rafn Methanolic extract	Red blood cells	STZ- diabetic rats	Induces CuZnSOD, CAT, GR activities; reduces TBARS and GSSP levels; increases GSH; reduces protein glycation and glycosylation; reduces blood glucose and HbA1C.	[25]

Abbreviations: HO-1, heme oxygenase-1; FBG, fasting blood glucose; HDL, high density lipoprotein; α2-M, α_2_-macroglobulin; IL-4, interleukin-4; IL-10, interleukin-10; RAGE, receptor for advanced glycation end-product; Nrf-2, nuclear factor erythroid 2-related factor 2; Mon./Macro.-like cells, Monocyte-/Macrophage-like cells.

Dietary intake of β-glucans (most commonly occurring in barley, oat, asparagus, maize, mushrooms, and fungi) was shown to reduce diabetes major risk factors such as hyperglycemia, hyperlipidemia, and hypertension. β-glucan-enriched extract (BGEE), containing (1,3/1,4) β-glucan with a small number of 1,6-linked glucose residues, administered to STZ-induced diabetic rats daily (80 mg/kg) for four weeks, improved diabetes-induced hyperglycemia, hyperlipidemia, and parameters that reflect liver and kidneys functionality in diabetes [48]. Detected increase in DNA damage and lipid peroxidation in the liver and kidneys of diabetic rats was significantly reduced by BGEE treatment, while the activities of CuZnSOD, MnSOD, and CAT, being reduced in diabetic condition in both organs, were found to be improved by BGEE treatment [48]. Described beneficial effects of BGEE on diabetic liver and kidneys could result from the reduced non-enzymatic glycation of proteins in serum and reduced O-glycosylation of MnSOD, CuZnSOD, and CAT antioxidant enzymes in the liver and kidney of diabetic rats [49]. BGEE treatment also attenuated inflammatory response in the liver of STZ-diabetic rats, evidenced by the normalization of acute-phase proteins in serum, as well as by the induction of mRNA expression of anti-inflammatory cytokines (interleukin (IL)-10 and IL-4) and inhibition of RAGE/NF-kB axis [10]. Together, these findings strongly support the therapeutic potential of BGEE in treating oxidative stress-induced pathological states such as diabetic condition. In addition to described effects of plant extracts, it has been found that flaxseed and fish oil could modulate expression of antioxidant and inflammatory genes thus contributing to reduction of liver inflammation and liver protein glycation in STZ-nicotinamide (NA) induced diabetic rats [50]. While a flaxseed oil diet induced activities and expression of CAT and SOD genes and protein level of GPx enzyme in liver of diabetic rats, a fish oil diet induced the expression and activity of CAT.

### 3.3. Plant Extracts Used in Human Clinical Trials

Positive effects of plant extracts demonstrated on experimental models of diabetes are further supported by human clinical trials (summarized in Table 3). T2D patients receiving hot water soaked *T. foenum graecum* seeds for eight weeks in small clinical trial, had significantly decreased fasting blood glucose, triglycerides, and LDL levels [51]. Long term (one year) treatment with *Nigella sativa* (powder in capsules of 500 mg) improved glucose homeostasis and enhanced antioxidant defense system in T2Dpatients under oral hypoglycemic therapy. In comparison to non-*Nigella sativa*-treated group of patients, a significant drop in fasting blood glucose (FBG), glycated hemoglobin (HbA1c) and TBARS levels and significant elevation of total antioxidant capacity were detected in *N. sativa*-treated patients. Moreover, cell response to insulin and the activity of β-cells were improved significantly after *N. sativa* treatment [52]. A single-blind randomized controlled clinical trial conducted on 64 T2D.

Patients (males and females; ages 30 to 60 years) revealed that brevis terminus intake of chamomile tea has a positive impact on glycemic control and antioxidant status. Consumption of chamomile tea (one cap (3 g/150 mL hot water), 3 times/day) for eight weeks lowered the level of HbA1c and MDA in serum and increased insulin sensitivity. In addition, total antioxidant capacity, SOD, GPx, and CAT activities in T2D patients were significantly increased by chamomile tea treatment [53]. A randomized controlled clinical study revealed the ability of *Salvia miltiorrhiza* hydrophilic extract (SMHE) to improve oxidative stress in diabetic patients with chronic heart disease. After two months of treatment with SMHE, the serum GSH level and activities of SOD, paraoxonase (PONase) and GR were markedly increased in diabetic patients receiving hypoglycemic therapy [54]. Another randomized human clinical trial revealed decrease in total and LDL cholesterol levels by soy protein without effects on HDL and triglycerides levels [55]. Protandim, a nutritional supplement consisting of extracts of five widely studied medicinal plants (*Withania somnifera, Bacopa monniera, Camellia sinensis, Silybum marianum,* and *Curcuma longa*) was shown to reduce plasma oxidative stress and to induce expression of endogenous SOD and CAT enzymes in healthy human subjects [56]. Vitamin complex (containing α-tocopherol ascorbic acid, β-carotene and) exerted dose dependent dual, pro-oxidant or antioxidant effects, in T1D patients. Low concentrations of vitamins in mix (ascorbic acid—0.08 µM, α-tocopherol—0.04 µM, β-carotene—0.0008 µM) were shown to reduce the expression of NADPH oxidase subunits, SOD and CAT genes in mononuclear cells of diabetic patients [57].

**Table 3:** Antidiabetic effects of plant extracts in clinical trials.

Plant Extract	Target	Model	Mechanism of Action	Reference
*Nigella sativa* (capsules/500 mg of powder)	Circulation Pancreas	T2D patients (oral hypoglycemic therapy)	Reduces FBG, HbA1c and TBARS; elevates TAC, SOD and GSH; improves cell response to insulin and the activity of β-cells.	[52]
*Matricaria chamomilla* Chamomile tea (3 g/150 mL hot water)	Circulation	T2D patients (non-insulin treatment)	Loweres HbA1c and MDA in serum and increases insulin sensitivity; increases SOD, GPx and CAT activities and TAC.	[53]
*Salvia miltiorrhiza* Hydrophilic extract	Circulation	Diabetic patients with chronic heart disease (oral hypoglycemic therapy)	Increases serum GSH level and activities of SOD, GR and PONase.	[54]
*Protandim* (nutritional supplement consisting of extracts of Bacopa monniera, Silybum marianum, Withania somnifera, Camellia sinensis and Curcuma longa)	Circulation	Healthy human subjects	Reduces oxidative stress and induces SOD and CAT expression in plasma.	[56]

Abbreviations: TAC, total antioxidant capacity; PONase, paraoxonase; SOD, superoxide dismutase; CAT, catalase; GPx, glutathione peroxidase; FBG, fasting blood glucose; HbA1c, glycated hemoglobin; TBARS, thiobarbituric acid reactive substances; GSH, glutathione; T2D, type 2 diabetes.

## 4. Protective Effects of Phytoconstituents in Diabetes Pathogenesis: Interaction with Antioxidant Gene Expression

Plants are an inexhaustible source of diverse biologically active compounds with potential to prevent or slow down islet failure, as well as diabetes-associated complications such as cardiovascular and liver disorders, nephropathy, and neuropathy. Those beneficial effects of phytoconstituents are achieved by stimulation of insulin secretion, modulation of carbohydrate metabolism and glucose uptake, decrease of cholesterol levels, antioxidant activity through direct or indirect elimination of free radicals and improvement of microcirculation [23]. Phytoconstituents with ameliorative effects on diabetes development and progression are presented in Table 4, Table 5 and Table 6.

### 4.1. Improvement of Insulin Secretion/Sensitivity and Glucose Homeostasis

Oleanolic acid (triterpenoid, abundant in Olive leaf) preserves viability and functionality of pancreatic islets, reduces insulin resistance, and protects against diabetic complications by repressing hyperlipidemia, advanced glycation end-products (AGEs) production andpolyol pathway, by stimulating PKB/Akt pathway, blocking Nf-κB and by inducing Nrf-2-mediated gene expression of GPx, SOD and phase II detoxification enzymes in β-cells, liver and kidney (reviewed in [58]). Daphnetin (7, 8-dihydroxycoumarin, isolated from cumarin contained in cinnamomum trees, green tea, carrots), exhibited insulin stimulating and antioxidant effects in rat insulinoma (INS-1) cells [59]. Pre-treatment of INS-1 cells with 1, 10, 20 and 40 µM of daphnetin for 24 h, followed by exposition to STZ (3 mM) for 12 h, improved cell viability and insulin secretion by reducing the levels of lipid peroxidation markers and improving activities of CAT, SOD, GPx and GST. Quercetin (flavonol, widely distributed in plant species, onions, grapes, citrus, berries, leafy vegetables, legumes, cocoa) has been shown to reduce blood glucose and improve plasma insulin level in STZ-diabetic BALB/c mice fed with quercetin containing diet for two weeks [60]. Quercetin diet also suppressed STZ-induced expression of cyclin-dependent kinase inhibitor p21(WAF1/Cip1) (Cdkn1a) and inducible nitric oxide (INOS2) genes in pancreas and liver of diabetic mice. Those findings suggest the ability of quercetin to improve pancreas and liver functioning by inducing proliferation/survival and regeneration of islet and liver cells. In another study, supplementation of T2D db/db mice with quercetin for six weeks, reduced plasma total cholesterol (TC) and increased HDL, lowered the level of TBARS and improved activities of CAT, SOD and GPx in the liver, indicating hypolipidemic and antioxidant effects of quercetin in T2D [61].

Curcumin (flavanoid, from *Curcuma longa*) decreased blood glucose level and improved insulin sensitivity in high fat diet (HFD) fed diabetic rats which could be attributed to anti-inflammatory and anti-lipolytic properties of curcumin, as evident by attenuation of free fatty acids and TNF-α levels in plasma [62]. Administration of curcumin dissolved in corn oil (15 mg/5 mL/kg body weight) for six weeks to STZ-diabetic rats, normalized their blood glucose and TBARS levels and increased concentration of GSH and the activities of CAT, SOD, GPx and GST antioxidant enzymes in the liver. Treatment of diabetic rats with curcumin exhibited significant increase in GST and SOD enzymes gene expressionindicating their regulation at the transcriptional level [63]. Phloridzin (dehydrochalcone present in apples and their derived products) given orally at various doses (5–40 mg/kg) to STZ-induced T1D rats has been shown to reduce post prandial hyperglycemia and improve dyslipidemia [64]. Berberine (alkaloid present in herbal plants such as *Berberis vulgaris* L., *Berberis aristata* L., *Coptis chinensis* Franch.) has been shown to regulate hyperglycemia and dyslipidemia in T2D patients [65] and to induce hepatic expression of CuZnSOD in STZ-NA diabetic mice [66]. Described effects are summarized in Table 4.

**Table 4:** Improvement of glucose homeostasis, β-cell viability and function by phytoconstituents.

Phytoconstituents and Their Sources	Effects	Type of Study	Mechanism of Action	Reference
**Oleanolic acid** (*Olea europaea* L.)	Increases insulin synthesis/secretion and improves glucose tolerance; promotes β-cell survival and proliferation.	INS-1 cells; STZ/Alloxan- diabetic mice/rats.	Induces Nrf-2-mediated gene expression of GPx, SOD and phase II enzymes and bocks Nf-κB; stimulates PKB/Akt pathway; represses polyol pathway, AGEs production and hyperlipidemia.	[58]
**Daphnetin** (Cinnamomum trees, green tea, carrots)	Improves β-cell viability and insulin secretion.	STZ-treated INS-1 cells	Reduces lipid peroxidation and improves SOD, CAT, GPx and GST activities.	[59]
**Quercetin** (Plant spices, onions, grapes, citrus, berries, leafy vegetables, legumes, cocoa)	Improves plasma insulin level; induces proliferation, survival and regeneration of islet and liver cells.	STZ-diabetic mice	Suppresses expression of Cdkn1a and INOS2.	[60]
**Curcumin** (*Curcuma longa*)	Improves insulin sensitivity; antilipolytic effects in plasma.	HFD diabetic rats	Attenuates TNF-α and free fatty acids levels in plasma.	[62]
**Phloridzin** (Apples and apple-derived products)	Antihyperglycemic, antihyperlipidemic effects.	STZ-diabetic rats	Reduces post prandial hyperglycemia and improves dyslipidemia.	[64]
**Berberine** (*Berberis aristata* L., *Berberis* vulgaris L., *Coptis chinensis* Franch.)	Regulates hyperglycemia and dyslipidemia.	T2D patients	Decreases fasting and postload plasma glucose, HbA1c, triglyceride, TC and LDL.	[65]

Abbreviations: Nrf-2, nuclear factor erythroid 2-related factor 2; SOD, superoxide dismutase; CAT, catalase; GPx, glutathione peroxidase; GST, glutathione S-transferase; Nf-κB, nuclear factor kappa B; AGEs, advanced glycation end-products; PKB/Akt, protein kinase B; Cdkn1a, cyclin-dependent kinase inhibitor; INOS2, inducible nitric oxide; TNF-α, tumor necrosis factor-α; LDL, low-density lipoprotein; TC, total cholesterol; HFD, high fat diet; HbA1c, glycated hemoglobin; T2D, type 2 diabetes.

### 4.2. Cardioprotective Effects of Phytoconstituents

Consumption of dietary polyphenols and flavonoids is inversely correlated with a development of cardiovascular risk factors such as glucose intolerance, dyslipidemia, and abdominal obesity [67,68]. Analyzing information from a prospective cohort study of 806 men (Zutphen Elderly Study), it was found that intake of catechin (flavonoid, the main component of tea) could be responsible for the inverse relation between tea consumption and ischemic heart disease [69]. Another phenolic compound, ellagic acid (EA), found in strawberries, raspberries, blackberries, cherries, and walnuts, also contributes to cardiovascular health. Ding and coworkers [70] reported that EA ameliorate atherosclerosis and oxidant-induced endothelial dysfunctionin apolipoprotein E-deficient (ApoE−/−) HFD C57BL/6 mice, by inducing nitric oxide synthase activity and antioxidant capacity in plasma. Additionally, EA increased Nrf-2 and HO-1 expression in the aortas and prevented hypochlorous acid (HOCl)-induced cellular damage. Treatment with quercetin attenuated cardiovascular remodeling and liver complications in HFD-induced metabolic syndrome in rats by lowering oxidative stress and inflammation through increased expression of Nrf-2, HO-1 and decreased expression of NF-kB [71]. Maslinic acid (triterpenoid, present in edible and medicinal plants) was shown to protect vascular smooth muscle cells (VSMCs) from increased ROS level via activation of Akt/Nrf-2 signaling pathway and by up-regulation of HO-1 expression [72]. Epigallo Catechin-3-O-Gallate (flavanol from green tea) was shown to lower protein and mRNA expression levels of the vascular cell adhesion molecules (VCAM1) and intercellular adhesion molecules (ICAM1) genes in human umbilical vein endothelial (HUVEC) cells [73]. Azafrin (carotenoid, from dried root of *Centranthera grandiflora*) is a highly present active compound in Chinese ethnodrug *Centranthera grandiflora* Benth. commonly used for treatment of cardiovascular diseases. Experimental evaluation of cardioprotective effects of azafrin revealed that it intensely improved cardiac function and reduced the infarct size in rats by decreasing levels of MDA and elevating SOD activity [74]. Such beneficial effects of azafrin could be also attributed to transcriptional activation of Nrf-2 and up-regulation of downstream target HO-1,

Thioredoxin-1 (Trx1), glutamate-cysteine ligase catalytic subunit (GCLC), NQO1, glutamate-cysteine ligase regulatory subunit (GCLM), and GST genes, demonstrated in human HEK293 embryonic kidney and H9c2 embryonic cardiomyocyte cell lines. Furthermore, cytosolic HO-1 and NQO1 and nuclear Nrf-2 protein levels were also found to be up-regulated in both in vivo and in vitro experiments. Another study revealed cardioprotective effect of triptolide (diterpenoid epoxide, from *Tripterygium wilfordii* Hook F) exerted by suppressed production of pro-inflammatory cytokines (TNF-α, IL-1β and IL-6) and induced activity of Nrf-2, SOD, GSH, GPx, and HO-1 enzymes in ischemic rat myocardium tissue [75]. Besides, α-Linolenic acid (ALA) (polyunsaturated fatty acid, from canola, soybean, wild berries, perilla and walnut), protected rats from doxorubicin (DOX)-induced cardiotoxicity by antioxidant and anti-apoptotic effects. ALA significantly eliminated DOX-induced decrease of myocardial SOD, GPx, and CAT content and further elevated mRNA levels of Nrf-2 and SOD [76].

Resveratrol (phytoalexin, especially present in red colored fruit such as grapes, peanuts, strawberries, cherries) possesses strong anti-inflammatory and antioxidant activities and thus has a potential for the prevention or treatment of diabetes pathogenesis [77]. Recent evidence indicates the ability of resveratrol to ameliorate cardiac oxidative stress and delay or diminish the progression of diabetes-related cardiac complications. Administration of resveratrol (50 mg/kg/day/orally, for 16 weeks) to HFD/STZ-induced T2D rats significantly reduced the levels of MDA and induced MnSOD activity, ATP content and mitochondrial membrane potential in diabetic hearts thus attenuating myocardial fibrosis and dysfunction in diabetes [78]. In another study, oral resveratrol administration to T2D rats (5 mg/kg/day for 4 months) induced cardiac SOD and CAT activities, reduced oxidative markers (oxidized/reduced GSH ratio, nitrite/nitrate, 8-isoprostane, NF-kB activity) and improved left ventricular pressure and coronary flow [79]. Orally administered resveratrol (2 mg/kg/day) for eight weeks to hypertensive STZ-induced diabetic rats increased serum NO availability, attenuated MDA, and interleukin (IL)-1β levels and improved vascular reactivity, left ventricular pump function and electrophysiology, demonstrating that resveratrol can elicit cardioprotective activity most probably via inhibition of Akt/NF-kB axis [80]. In addition, resveratrol has been shown to contribute to cardiovascular protection by activating of Nrf-2 and by enhancing the expression of SOD, GPx1, Trx-1, Trx-2, glutaredoxin (Grx)-1, Grx-2, HO-1, NQO1, NQO2 and GCLC, the rate-limiting enzyme for GSH synthesis (reviewed in [81]).

Endothelial inflammation is a risk factor in the pathogenesis of majority of cardiovascular diseases. Bioactive compound (−)-7(S)-hydroxymatairesinol (7-HMR), naturally occurring plant lignan from *Picea abies* (Norway spruce) inhibited TNF-α-stimulated endothelial inflammation in rat aortic endothelial cells by lowering NF-κB activity, attenuating generation of ROS and up-regulating the expression of Nrf-2 and its target SOD and HO-1 genes [82]. Baicalein (flavone, from *Scutellaria baicalensis* and *Scutellaria lateriflora*), used as a food supplement, is able to attenuate diabetes and its complications by reducing ROS levels and by increasing gene expression and activities of antioxidant enzymes in diabetic rats. Thus, administration of baicalein alleviated DOX-induced cardiotoxicity in BALB/c mice via suppression of myocardial oxidative stress and increased myocardial expression of Nrf-2 and HO-1 [83]. In addition, baicalein application to STZ-NA diabetic rats significantly lowered the blood glucose and HbA1c levels, and increased activities and expression of CAT, SOD, GPx and GSH in the liver, which was even more efficient than in glibenclamide-treated diabetic rats [84]. Curcumin (flavanoid, from *Curcuma longa*) is known to have multiple bioactivities including cardioprotection. Using an embryonic cardiomyocyte cell line and HFD-mice model, Zeng and coworkers [85] demonstrated that curcumin suppressed oxidative stress and inflammation by activating HO-1, Nrf-2 and NQO-1 and inactivating NF-kB. Sulforaphane (1-isothiocyanate-4-methylsulfinylbutane, from broccoli sprouts) was analyzed for its ameliorating effects in diabetic complications. In a double-blind clinical trial of T2D patients, sulforaphane demonstrated a significant improvement of insulin resistance [86]. Also, prolonged (3 months or 6 months) treatment of STZ-induced diabetic mice with sulforaphane significantly activated Nrf-2 signaling and mRNA expression of NQO1, HO-1, metallothionein (MT), SOD1, SOD2 and CAT in the heart of diabetic mice and prevented diabetes-induced cardiac oxidative damage, hypertrophy, inflammation, and fibrosis [87]. Other studies also revealed that protective effects of sulforaphane on diabetic cardiomyopathy and nephropathy are mediated by MT, a downstream target of Nrf-2 [88,89]. Cardioprotective effects of phytoconstituents are summarized in Table 6.

### 4.3. Kidney and Liver Protection by Phytoconstituents

Pathogenesis of diabetic nephropathy is directly linked with hyperglycemia-induced proximal tubule injury. Zhou and coworkers [90] reported renoprotective potential of obacunone, triterpenoid limonoid compound present mainly in citrus and other plants of the Rutaceae family. Namely, obacunone prevented high glucose (HG)-induced oxidative damage of renal tubular epithelial cells (NRK-52E) by increasing the levels of antioxidant enzymes (CAT, SOD, and GSH), inhibiting ROS production and stabilizing the mitochondrial membrane potential. Additionally, obacunone down-regulated activity of glycogen synthase kinase 3 beta (GSK-3β) and up-regulated activity of Nrf-2, thus enhancing mRNA expression of its target NQO-1 and HO-1 genes in HG-treated cells. Evaluation of the effect of resveratrol on renal tissue in STZ-diabetic rats revealed that resveratrol may confer beneficial effects on kidney functions through modulation of antioxidant enzymes level and insulin signaling [91]. Diabetes-induced reduction of CAT and increase of SOD1 protein levels in rat kidney tissue were normalized by resveratrol toward the control values, whereas down-regulated mRNA levels of CAT, GPx and SOD1 were not affected by resveratrol treatment. In addition, resveratrol application increased protein level of SOD2 in both control and diabetic groups, suggesting involvement of resveratrol in post-translational regulation of CAT, and gene expression. Another natural plant resource for diabetes therapy, saponins from *Gynostemma pentaphyllum* (GPs) ameliorated hyperglycemia, dyslipidemia, and significantly increased insulin levels in STZ-induced diabetic rats [92]. In addition, GPs treatment promoted expression of Nrf-2 in the liver of diabetic rats stimulating significant increase in the activities of SOD and GPx in comparison to control animals. Increased activities of SOD and GPx were also detected in kidneys of GPs-treated diabetic rats.

**Table 5:** Phytoconstituents—gene interactions in attenuation of diabetic liver and kidney disorders.

Phytoconstituents and Their Sources	Effects	Type of Study	Mechanism of Action	Reference
**Quercetin** (Plant spices, onions, grapes, citrus, berries, leafy vegetables, legumes, cocoa)	Reduces TC and increases HDL; liver antioxidant protection. Attenuates liver complications.	T2D db/db mice HFD- metabolic syndrome in rats	Lowers TBARS and improves liver SOD, CAT and GPx activities. Increases liver Nrf-2, HO-1 and decreases NF-kB expression.	[61] [71]
**Curcumin** (*Curcuma longa*)	Normalizes blood glucose; improves antioxidant protection in liver.	STZ-diabetic rats	Induces hepatic GSH level, SOD, CAT, GPx, GST activities and SOD and GST expression.	[63]
**Berberine** (*Berberis aristata* L., Berberis vulgaris L., *Coptis chinensis* Franch.)	Hypoglycemic effect; liver protection.	STZ-NA diabetic mice	Induces hepatic expression of CuZnSOD.	[66]
**Baicalein** (Scutellaria baicalensis, S. lateriflora)	Reduced diabetes-related oxidative stress in liver.	STZ-NA diabetic rats	Lowers blood glucose and HbA1c; increases activities and expression of SOD, CAT, GSH and GPx in the liver.	[84]
**Obacunone** (citrus and plants of the Rutaceae family)	Renoprotective effect by preventing HG-induced oxidative damage of renal tubular epithelial cells.	HG-treated NRK-52E cells	Increases SOD, GSH, CAT levels; down-regulates activity of GSK-3β and up-regulates activity of Nrf-2; enhances mRNA of NQO-1 and HO-1 genes.	[90]
**Resveratrol** (grapes, peanuts, strawberries, cherries)	Beneficial effect on kidney function.	STZ-diabetic rats	Normalizes CAT, SOD1 and SOD2 protein levels in kidneys toward control values.	[91]
**Saponins** (*Gynostemma pentaphyllum*)	Ameliorates hyperglycemia, dyslipidemia and insulin levels. Renal- and hepato-protection.	STZ-diabetic rats	Promotes Nrf-2 expression and SOD and GPx activities in the liver; increases kidney SOD and GPx activities.	[92]
**Alpha-lipoic acid** (spinach, broccoli, tomato, carrots)	Decreases glycosylation of antioxidant and redox signaling proteins in diabetic liver, kidney and circulation.	STZ- diabetic rats	Improves glucose, triglycerides, HbA1c, AST and ALT in serum; elevates GSH level; induces activities of SOD, CAT and reduces SOD, CAT, HSP70, HSP90 glycosylation in RBCs.	[93]
		STZ- diabetic rat kidney	Promotes activities of renal MnSOD, CuZnSOD and CAT by inducing their mRNA levels and by reducing their glycosylation.	[94]
		STZ- diabetic rat liver	Restores CAT and Mn/CuZnSOD activities; increases mRNA and protein levels of CuZnSOD and CAT; decreases glycosylation of SOD, CAT, ERK, p38 NFkB-p65, CEBPβ in liver.	[16]

Abbreviations: Nrf-2, nuclear factor erythroid 2-related factor 2; SOD, superoxide dismutase; CAT, catalase; GPx, glutathione peroxidase; GSH, glutathione; GST, glutathione S-transferase; HDL, high density lipoprotein; TC, total cholesterol; HFD, high fat diet; NA, nicotinamide; HO-1, heme oxygenase-1; NQO1, nicotinamide adenine dinucleotide phosphate quinone oxidoreductase 1; HG, high glucose; HbA1c, glycated hemoglobin; HSP70 and HSP90, heat shock proteins 70 and 90; GSK-3β, glycogen synthase kinase-3 beta; RBC, red blood cells; ERK, extracellular signal-regulated kinase; p38, mitogen-activated protein kinase; NFkB-p65, nuclear factor kappa B protein subunit 65; CEBPβ, CCAAT/enhancer-binding protein beta; ALT, alanine aminotransferase; AST, aspartate aminotransferase; NRK-52E cells, rat kidney epithelial cells.

Alpha-lipoic acid (LA) (dithiol compound found in spinach, broccoli, tomato, carrots), is a therapeutic agent applied for the treatment of diabetic retinopathy and neuropathy due to its metal-chelating and free radical scavenging activities. Decreased glycosylation of the key proteins, involved inantioxidant protection and redox signaling pathways in diabetes was suggested as an additional mechanism of the antioxidant effect of LA [16,93,94]. This finding is important considering that hyperglycemia-promoted post-translational addition of beta-N-acetylglucosamine (O-GlcNAc) to proteins plays important role in diabetes etiology and pathology. Administration of LA to STZ-induced diabetic rats (10 mg/kg that equals to 600 mg LA/day in humans) for 4 weeks resulted with improved glucose, glycated hemoglobin, triglycerides levels, as well as serum activities of aspartate aminotransferase (AST) and alanine aminotransferase (ALT) [93]. In addition, administration of LA to diabetic rats elevated levels of GSH and enhanced activities of SOD and CAT by alleviating their and glycosylation of heat shock proteins (HSP70 and HSP90) in RBCs. Such protective effect of LA on structure and stability of RBC proteins could inhibit or delay diabetic complications. In accordance, LA administration to STZ-diabetic rats activated cytoprotective response against diabetes-induced oxidative injury in kidneys [94]. LA positively influenced activities of renal CAT, MnSOD and CuZnSOD enzymes by inducing their mRNA levels and by reducing their glycosylation. LA also lowered oxidative stress in diabetic rat liver and restored enzymatic activities of CuZnSOD, MnSOD and CAT, by affecting their expression at the transcriptional and the post-transcriptional level [16]. Administration of LA up-regulated mRNA and protein expression of CuZnSOD and CAT and reduced glycosylation of SOD, CAT, and their gene regulatory proteins (ERK and p38 kinase, NF-kB-p65, CEBPβ) in STZ-diabetic rat liver. Kidney and liver protection by phytoconstituents is summarized in Table 5.

### 4.4. Impact of Phytoconstituents on Epigenetic Regulation

Dietary-induced epigenetic changes may also influence gene expression of antioxidant enzymes. Different phytoconstituents (such as sulforaphane, quercetin, curcumin, and reserpine) have been shown to induce Nrf-2 signaling through epigenetic mechanisms [95]. In vitro and in vivo treatment with dioscin (steroid saponin from *Dioscorea nipponica* Makino) regulated the intracellular levels of MDA, ROS, SOD, GPx, and GSH and thereby significantly inhibited myocardial oxidative insult [96]. In addition, dioscin application was able to activate Nrf-2 and histone deacetylase Sirt2 signaling pathways that in turn affected the expression of NQO1, HO-1, GCLM, GST, kelch-like ECH-associated protein 1 (Keap1) and FOXO3a through decreased expression of miR-140-5p level in H9c2 embryonic cardiomyocyte cell line [96]. Curcumin was also demonstrated to activate antioxidant cellular defense by introducing epigenetic changes in the Nrf-2 gene. Curcumin-mediated demethylation of Nrf-2 gene was found to be associated with up-regulation of Nrf-2 and its target NQO1 gene at the mRNA and protein levels in TRAMP C1 prostate cancer cells [15]. Recent evidence revealed that resveratrol diminishes cardiac injury in HFD/STZ-induced diabetic rats through Sirt1 activation, which in turn partially contributes to the regulation of mitochondrial function [78]. Resveratrol promotes endothelium-dependent vascular relaxation and improves endothelial function by inducing Sirt1-mediated deacetylation of endothelial nitric oxide synthase (eNOS) and endothelial NO bioactivity [97]. Similarly, in T2D patients with coronary artery disease and hypertension, 12 months treatment with grape extract containing 8 mg of resveratrol lowered the expression of proinflammatory IL-1β and TNF-α cytokines by mediating activity of inflammation-related miRNAs (miR-181b, miR-21, miR-30c2, miR-34a, miR-155 and miR-663) [98]. These data (summarized in Table 6) indicate the importance of further elucidation of the involvement of epigenetic mechanisms in antioxidant and antidiabetic effects of natural products.

**Table 6:** Phytoconstituents attenuate diabetic cardiovascular complications.

Phytoconstituents and Their Sources	Effects	Type of Study	Mechanism of Action	Reference
**Quercetin** (Plant spices, onions, grapes, citrus, berries, leafy vegetables, legumes, cocoa)	Attenuated cardiovascular complications.	HFD-induced metabolic syndrome in rats	Increases Nrf-2, HO-1 and decreases NF-kB expression in heart.	[71]
**Curcumin** (*Curcuma longa*)	Cardioprotection.	HFD-mice model	Activates Nrf-2, HO-1, NQO-1 and inactivates NF-kB.	[85]
**Catechin** (main component of tea)	Inversed relation with ischemic heart disease mortality.	Zutphen Elderly Study of men aged 65–84 years	Reduces the risk of ischemic heart disease.	[69]
**Ellagic acid** (Strawberries, raspberries, blackberries, cherries, walnuts)	Improved oxidant-induced endothelial dysfunction and atherosclerosis.	HFD- ApoE (−/−) C57BL/6 mice	Induces NO synthase activity and antioxidant capacity in plasma; increases Nrf-2 and HO-1 expression in aortas; prevents HOCl-induced cellular damage.	[70]
**Maslinic acid** (Medicinal plants)	Protection of VSMCs from oxidative stress.	VSMCs from Sprague-Dawley rats	Activates Akt/Nrf-2 signaling pathway and up-regulates expression of HO-1.	[72]
**Epigallo Catechin-3-O-Gallate** (Green tea)	Atherosclerosis protection.	HUVEC cells	Loweres mRNA and protein expression of VCAM1 and ICAM1 genes.	[73]
**Azafrin** (*Centranthera grandiflora* Benth.)	Cardioprotection.	MI/MIR injured rats; HEK293 and H9c2 cell lines	Lowers MDA and elevates SOD activity in serum; increases protein levels of HO-1, NQO1, Nrf-2; up-regulates Nrf-2, HO-1, NQO1, GCLC, GCLM, Trx1 and GST gene expression.	[74]
**α-Linolenic acid** (canola, soybean, wild berries)	Cardioprotection.	DOX-induced cardiotoxicity in rats	Elevates mRNA level of myocardial Nrf-2 and SOD.	[76]
**7-HMR** (Picea abies)	Inhibition of endothelial inflammation.	Rat aortic endothelial cells	Induces Nrf-2, SOD and HO-1 gene expression.	[82]
**Baicalein** (*Scutellaria baicalensis*, *S. lateriflora*)	Reduced myocardial oxidative stress.	DOX-treated BALB/c mice	Increases Nrf-2 and HO-1 myocardial expression.	[83]
**Triptolide** (*Tripterygium wilfordii* Hook F)	Cardioprotection.	Ischemic (I/R) rats	Suppresses TNF-α, IL-1β, IL-6 production and induces Nrf-2, GSH, SOD, GPx, HO-1 activity in ischemic myocardium tissue.	[75]
**Sulforaphane** (broccoli sprouts)	Prevents diabetic cardiac oxidative damage and dysfunction.	STZ-induced diabetic mice	Activates cardiac Nrf-2 signaling and mRNA/protein levels of HO-1, NQO1, MT, CAT, SOD1, SOD2.	[87]
**Resveratrol** (grapes, peanuts, strawberries, cherries)	Attenuates cardiac oxidative stress and complications. Attenuates cardiac oxidative insult through epigenetic regulation. Improves coronary artery disease through epigenetic regulation.	HFD/STZ-T2D rats HFD/STZ- diabetic rats T2D patients	Reduces MDA and induces MnSOD activity in heart; Sirt1 activation. Lowers IL-1β and TNF-α expression by mediating activity of miRNAs.	[78] [78] [98]
**Dioscin** (*Dioscorea nipponica* Makino)	Protection against myocardial oxidative insult through epigenetic regulation.	H9c2 cell line	Activates Nrf-2 and Sirt2 signaling; induces expression of HO-1, NQO1, GST, GCLM, Keap1 and FOXO3a; decreases expression of miR-140-5p in cardiomyocytes.	[96]

Abbreviations: HFD, high fat diet; ApoE (−/−), apolipoprotein E-deficiency; NO, nitric oxide; HOCl, hypochlorous acid; VSMCs, vascular smooth muscle cells; VCAM1, vascular cell adhesion molecules; ICAM1, intercellular adhesion molecules; HUVEC cells, human umbilical vein endothelial cells;. GCLC, glutamate-cysteine ligase catalytic subunit; GCLM, glutamate-cysteine ligase regulatory subunit; Trx1, thioredoxin-1; HEK293, human embryonic kidney cell line; H9c2, embryonic cardiomyocyte cell line; MI, myocardial infarction; MIR, myocardial ischemia-reperfusion; I/R, ischemia/reperfusion injuries; DOX, doxorubicin; 7-HMR, (−)-7(S)-hydroxymatairesinol; MT, metallothionein; Keap1, kelch-like ECH-associated protein 1; FOXO3a, forkhead box O3; Sirt2, silent information regulators; miR-140-5p, microRNA 140-5p.

## 5. Long Journey from Phytochemical Composition to Biological Activity and Human Consumption

Use of plant extracts and phytoconstituents has become more prominent at present for the prevention or treatment of different health problems. The contribution of herbal medicine in the general public health raises problems associated with classification of many of these products as foods or dietary supplements that do not require evidence of safety, quality, and efficacy before they reach the market [99]. Therefore, the use of plant extracts or phytoconstituents, in parallel with conventional drugs, certainly requires a product license that has to include safety measures, quality control, and efficacy data [100].

Identifying the bioavailability of bioactive plant extracts/food compounds is essential for evaluation of their potential health benefits. Bioavailability of plant extracts or phytoconstituents is the most important for their full efficacy in organism and implies deliverance, absorption, distribution, metabolism, and elimination phases of extract/constituent [101]. In addition, many crude plant extracts or phytoconstituents demonstrated good biological activities (e.g., antioxidant activities) in in vitro assays, while slightly reduced activity was determined in in vivo studies. One of the main reasons for more efficient in vitro effect of plant extracts or phytoconstituents lies in used effective concentrations that are usually higher in comparison to the concentration (dose) used for in vivo assays. Applied in vivo, after consumption, distribution and metabolic degradation, the effective concentration that reaches target tissue or organ and display biological reaction is far less concentrated if compared to in vitro tested [102]. Therefore, several technologies have been made trying to solve or enhanced bioavailability including structural modifications, nanotechnology, and colloidal systems [103]. It should be also be born in mind that in addition to the above-mentioned, pharmacokinetics of convinced compounds may be also influenced by the gender, age, and pathological status of the host. One of the main directions of recent decades in the research and application of herbal medicine was separation strategy and obtaining pure component from the extract with the aim of increasing bioavailability of active plant extract compound. Conversely, several studies revealed that the pharmacological effects of many bioactive constituents decline when compared to their action from the crude extracts [104]. It was shown that some coexisting constituents (such as plant primary and secondary metabolites) promote enhanced bioavailability in organism or change the forms of bioactive constituents (including the formation of inner natural nanoparticles) making crude extracts as a mixture of bioactive compounds and pharmacokinetic synergists [104].

Testing the bioavailability of plant extracts or phytoconstituents is very important for defining their potential health benefits, but at the same time allows evaluation of their undesired toxic side effects. In general, this includes establishing the exposure concentration/dose at which certain adverse effect are observed. A substantial effort has been made to synchronize methods for toxicity testing that can be used for each herbal medicine that will be introduced for human consumption [105]. Tests for toxicological characterization encompass chronic low-dose toxicity tests, tests for acute high-dose exposure effects, and specific cellular, organ and system-based toxicity assays [106].

Taken together, all plant extract/phytonstituents investigations as regards their use in prevention and treatment of diseases and human consumption require intensive studies with emphasis on numerous factors such as bioavailability, the internal duration, the curative amount reaching the target tissue, combination/interactions with other drugs or herbs and other pharmacokinetic parameters of plant extracts and bioactive compounds as well as human population age, gender and pathological status.

## 6. Conclusions

Extensive in vitro and in vivo experimental investigations of plants and their phytoconstituents provided promising outcomes in attenuating diabetes and its complications. Reported blood glucose lowering and multileveled antioxidative effects of natural products are further supported by clinical trials with diabetic patients giving a great potential for the future clinical use of bioactive compounds in the prevention or treatment of diabetes. Medicinal plants and diverse plant-derived biologically active compounds have the potential to alleviate the symptoms of diabetes by preventing or slowing down islet failure and diabetes-associated complications such as cardiovascular and liver disorders, nephropathy, and neuropathy. Those beneficial effects of phytoconstituents are achieved through stimulation of insulin secretion, modulation of carbohydrate metabolism and glucose uptake, decrease of cholesterol levels, antioxidant activity through direct or indirect elimination of free radicals and improvement of microcirculation. However, potential clinical use of natural products in diabetes management requires extensive studies on their bioavailability, specificity, and underlying mechanisms of action, including nutrigenomic approach and in-depth analysis of phytoconstituents-gene interaction. Elucidation of such mechanisms could provide basis for the development of future high-selective antioxidant compounds for diabetes management. At the same time, through these mechanisms of action plant-derived compounds could act preventively and lower the risk of developing diabetes. The summarized effects of phytoconstituents on the antioxidant enzymes’ gene expression represent additional effort for pointing out the importance of nutrition impact on human health.

## Abbreviations

STZ, streptozotocin; SNP, sodium nitroprusside; AAPH, 2,2-azo-bis(2-amidinopropane)dihydrochloride; SOD, superoxide dismutase; CAT, catalase; GPx, glutathione peroxidase; GR, glutathione reductase; GST, glutathione S-transferase; GSSP, glutathione disulfide; MDA, malondialdehyde; TBARS, thiobarbituric acid reactive substances; GSH, glutathione; -SH, sulfhydryl group; NF-kB, nuclear factor kappa B; Nrf-2, nuclear factor erythroid 2-related factor 2; GLUT-2, glucose transporter 2; PKB/Akt, protein kinase B; ERK, extracellular signal-regulated kinase; p38, mitogen-activated protein kinase; Pdx-1, pancreatic and duodenal homeobox 1; MafA, musculoaponeurotic fibrosarcoma oncogene homolog A; H_2_O_2_, hydrogen peroxide; Rin-5F, rat pancreatic cell line; HO-1, heme oxygenase-1; FBG, fasting blood glucose; HbA1c, glycated hemoglobin; AGEs, advanced glycation end-products; RAGE, receptor for advanced glycation end-product; LDL, low-density lipoprotein; HDL, high density lipoprotein; α2-M, α_2_-macroglobulin; TNF-α, tumour necrosis factor-α; IL-6 interleukin-6; IL-1β, interleukin-1β; IL-4, interleukin-4; IL-10, interleukin-10; Mon./Macro.-like cells, Monocyte-/Macrophage-like cells; TAC, total antioxidant capacity; PONase, paraoxonase; T1D, type 2 diabetes; T2D, type 2 diabetes; TC, total cholesterol; Cdkn1a, cyclin-dependent kinase inhibitor; INOS2, inducible nitric oxide; HFD, high fat diet; ApoE (−/−), apolipoprotein E-deficiency; NO, nitric oxide; HOCl, hypochlorous acid; VSMCs, vascular smooth muscle cells; VCAM1, vascular cell adhesion molecules; ICAM1, intercellular adhesion molecules; HUVEC cells, human umbilical vein endothelial cells; NQO1, nicotinamide adenine dinucleotide phosphate quinone oxidoreductase 1; GCLC, glutamate-cysteine ligase catalytic subunit; GCLM, glutamate-cysteine ligase regulatory subunit; Trx1, thioredoxin-1; HEK293, human embryonic kidney cell line; H9c2, embryonic cardiomyocyte cell line; MI, myocardial infarction; MIR, myocardial ischaemia-reperfusion; I/R, ischemia/reperfusion injuries; DOX, doxorubicin; 7-HMR, (−)-7(S)-hydroxymatairesinol; MT, metallothionein; Keap1, kelch-like ECH-associated protein 1; FOXO3a, forkhead box O3; Sirt2, silent information regulators; miR-140-5p, microRNA 140-5p; NA, nicotinamide; HSP70 and HSP90, heat shock proteins 70 and 90; GSK-3β, glycogen synthase kinase-3 beta; RBC, red blood cells; NFkB-p65, nuclear factor kappa B protein subunit 65; CEBPβ, CCAAT/enhancer-binding protein beta; ALT, alanine aminotransferase; AST, aspartate aminotransferase; NRK-52E cells, rat kidney epithelial cells.

## References

[1] Berná G., Oliveras-López M.J., Jurado-Ruíz E., Tejedo J., Bedoya F., Soria B., Martín F. (2014). Nutrigenetics and Nutrigenomics Insights into Diabetes Etiopathogenesis. Nutrition.

[2] Karuranga S., Fernandes J., Huang Y., Malanda B. (2017). IDF Diabetes Atlas 2017.

[3] Ren B., O’Brien B.A., Swan M.A., Koina M.E., Nassif N., Wei M.Q., Simpson A.M. (2007). Long-term correction of diabetes in rats after lentiviral hepatic insulin gene therapy. Diabetologia.

[4] Nasri H., Rafieian-Kopaei M. (2014). Metformin: Current knowledge. J. Res. Med. Sci..

[5] Liu Z., Zhao X., Sun W., Wang Y., Liu S., Kang L. (2017). Metformin combined with acarbose vs. single medicine in the treatment of type 2 diabetes: A meta-analysis. Exp. Ther. Med..

[6] Subbulakshmi G., Naik M. (2001). Indigenous foods in the treatment of diabetes mellitus. Bombay Hosp. J..

[7] Jarald E., Joshi S.B., Jain D.C. (2008). Diabetes vs. Herbal Medicines. Iranian J. Pharmacol. Therap..

[8] Sharma B., Mittal A., Dabur R. (2018). Mechanistic approach of anti-diabetic compounds identified from natural sources. Chem. Biol. Lett..

[9] Schofield C.J., Sutherland C. (2012). Disordered insulin secretion in the development of insulin resistance and Type 2 diabetes. Diabet. Med..

[10] Uskoković A., Mihailović M., Dinić S., Arambašić-Jovanović J., Grdović N., Marković J., Poznanović G., Vidaković M. (2013). Administration of a beta-glucan-enriched extract activates beneficial hepatic antioxidant and anti-inflammatory mechanisms in streptozotocin-induced diabetic rats. J. Funct. Foods.

[11] Valko M., Leibfritz D., Moncol J., Cronin M.T.D., Mazur M., Telser J. (2007). Free radicals and antioxidants in normal physiological functions and human disease. Int. J. Biochem. Cell. Biol..

[12] Radi R. (2018). Oxygen radicals, nitric oxide, and peroxynitrite: Redox pathways in molecular medicine. Proc. Natl. Acad. Sci. USA.

[13] Matés J.M. (2000). Effects of antioxidant enzymes in the molecular control of reactive oxygen species toxicology. Toxicology.

[14] Keum Y.S., Han Y.H., Liew C., Kim J.H., Xu C., Yuan X., Shakarjian M.P., Chong S., Kong A.N. (2006). Induction of heme oxy-genase-1 (HO-1) and NAD[P]H: Quinone oxidoreductase 1 (NQO1) by a phenolic antioxidant, butylated hydroxyanisole (BHA) and its metabolite, tert-butylhydroquinone (tBHQ) in primary-cultured human and rat hepatocytes. Pharm. Res..

[15] Khor T.O., Huang Y., Wu T.-Y., Shu L., Lee J., Kong A.-N.T. (2011). Pharmacodynamics of curcumin as DNA hypomethylation agent in restoring the expression of Nrf2 via promoter CpGs demethylation. Biochem. Pharmacol..

[16] Dinić S., Arambašić J., Mihailović M., Uskoković A., Grdović N., Marković J., Karadžić B., Poznanović G., Vidaković M. (2013). Decreased O-GlcNAcylation of the key proteins in kinase and redox signalling pathways is a novel mechanism of the bene-ficial effect of alpha-lipoic acid in diabetic liver. Br. J. Nutr..

[17] Pavlidis C., Patrinos G.P., Katsila T. (2015). Nutrigenomics: A controversy. Appl. Transl. Genom..

[18] Bhatti F., Mankhey R.W., Asico L., Quinn M.T., Welch W.J., Maric C. (2005). Mechanisms of antioxidant and pro-oxidant effects of alphalipoic acid in the diabetic and non-diabetic kidney. Kidney Int..

[19] Lapolla A., Traldi P., Fedele D. (2005). Importance of measuring products of non-enzymatic glycation of proteins. Clin. Biochem..

[20] Brownlee M. (2001). Biochemistry and molecular cell biology of diabetic complications. Nat. Cell Biol..

[21] Chung S.S., Ho E.C., Lam K.S., Chung S.K. (2003). Contribution of Polyol Pathway to Diabetes-Induced Oxidative Stress. J. Am. Soc. Nephrol..

[22] Unuofin J.O., Lebelo S.L. (2020). Antioxidant Effects and Mechanisms of Medicinal Plants and Their Bioactive Compounds for the Prevention and Treatment of Type 2 Diabetes: An Updated Review. Oxid. Med. Cell. Longev..

[23] Bharti S.K., Krishnan S., Kumar A., Kumar A. (2018). Antidiabetic phytoconstituents and their mode of action on metabolic pathways. Ther. Adv. Endocrinol. Metab..

[24] Sefi M., Fetoui H., Lachkar N., Tahraoui A., Lyoussi B., Boudawara T., Zeghal N. (2011). *Centaurium erythrea* (Gentianaceae) leaf extract alleviates streptozotocin-induced oxidative stress and β-cell damage in rat pancreas. J. Ethnopharmacol..

[25] Đorđević M., Mihailović M., Jovanović J.A., Grdović N., Uskoković A., Tolić A., Sinadinović M., Rajić J., Mišić D., Šiler B. (2017). Centaurium erythraea methanol extract protects red blood cells from oxidative damage in streptozotocin-induced diabetic rats. J. Ethnopharmacol..

[26] Đorđević M., Grdović N., Mihailović M., Jovanović J.A., Uskoković A., Rajić J., Sinadinović M., Tolić A., Mišić D., Šiler B. (2019). *Centaurium erythraea* extract improves survival and functionality of pancreatic beta-cells in diabetes through multiple routes of action. J. Ethnopharmacol..

[27] Đorđević M., Grdović N., Mihailović M., Jovanović J.A., Uskoković A., Rajić J., Đorđević M., Tolić A., Mišić D., Šiler B. (2020). *Centaurium erythraea* extract reduces redox imbalance and improves insulin expression and secretion in pancreatic β-cells exposed to oxidative and nitrosative stress. Arch. Biol. Sci..

[28] Narváez-Mastache J.M., Soto C., Delgado G. (2007). Antioxidant evaluation of Eysenhardtia species (Fabaceae): Relay synthesis of 3-O-Acetyl-11alpha,12alpha-epoxy-oleanan-28,13beta-olide isolated from *E. platycarpa* and its protective effect in experimental diabetes. Biol. Pharm. Bull..

[29] Mujić A., Grdović N., Mujić I., Mihailović M., Živković J., Poznanović G., Vidaković M. (2011). Antioxidative effects of phenolic extracts from chestnut leaves, catkins and spiny burs in streptozotocin-treated rat pancreatic β-cells. Food Chem..

[30] Grdović N., Dinić S., Arambašić J., Mihailović M., Uskoković A., Marković J., Poznanović G., Vidović S., Zeković Z., Mujić A. (2012). Protective effect of Lactarius deterrimus and Castanea sativa extract mix (MIX Ld/Cs) on STZ-induced oxidative stress and pancreatic beta-cell death. Br. J. Nutr..

[31] Gandhi G.R., Ignacimuthu S., Paulraj M.G. (2011). *Solanum torvum* Swartz. fruit containing phenolic compounds shows antidiabetic and antioxidant effects in streptozotocin induced diabetic rats. Food Chem. Toxicol..

[32] Arambašić Jovanović J., Mihailović M., Uskoković A., Grdović N., Dinić S., Poznanović G., Mujić I., Vidaković M. (2017). Evalu-action of the antioxidant and antiglycation effects of *Lactarius deterrimus* and *Castanea sativa* extracts on hepatorenal injury in streptozotocin-induced diabetic rats. Front. Pharmacol..

[33] Mihailović M., Uskoković A., Jovanović J.A., Grdović N., Dinić S., Poznanović G., Franić A., Đorđević M., Vidaković M. (2020). Treatment of streptozotocin-induced diabetic rats with *Castanea sativa* and *Lactarius deterrimus* extracts decreases liver damage by initiating activation of the Akt prosurvival kinase. Arch. Biol. Sci..

[34] Liu Y., Chen P., Zhou M., Wang T., Fang S., Shang X., Fu X. (2018). Geographic variation in the chemical composition and anti-oxidant properties of phenolic compounds from *Cyclocarya paliurus* (Batal) Iljinskaja leaves. Molecules.

[35] Alam M., Kwon K., Lee S., Lee S. (2017). *Lannea coromandelica* (Houtt.) Merr. induces heme oxygenase 1 (HO-1) expression and reduces oxidative stress via the p38/c-Jun N-terminal kinase-nuclear factor erythroid 2-related factor 2 (p38/JNK-NRF2)-mediated antioxidant pathway. Int. J. Mol. Sci..

[36] Shukla R., Banerjee S., Tripathi Y.B. (2018). Antioxidant and Antiapoptotic effect of aqueous extract of *Pueraria tuberosa* (Roxb. Ex Willd.) DC. On streptozotocin-induced diabetic nephropathy in rats. BMC Complement. Altern. Med..

[37] Ojo O.A., Okesola M.A., Ekakitie L.I., Ajiboye B.O., Oyinloye B.E., Agboinghale P.E., Onikanni A.S., Lisa E. (2020). *Gongronema latifolium* Benth. leaf extract attenuates diabetes-induced neuropathy via inhibition of cognitive, oxidative stress and inflammatory response. J. Sci. Food Agric..

[38] Miaffo D., Kamgue O., Guessom T., Temhoul C., Kamanyi A. (2019). Antidiabetic and antioxidant potentials of Vitellaria para-doxa barks in alloxan-induced diabetic rats. Clin. Phytosci..

[39] Sasidharan I., Sundaresan A., Nisha V.M., Kirishna M.S., Raghu K.G., Jayamurthy P. (2012). Inhibitory effect of *Terminalia chebula* Retz. fruit extracts on digestive enzyme related to diabetes and oxidative stress. J. Enzym. Inhib. Med. Chem..

[40] Justino A.B., Pereira M.N., Peixoto L.G., Vilela D.D., Caixeta D.C., De Souza A.V., Teixeira R.R., Silva H.C.G., De Moura F.B.R., Moraes I.B. (2017). Hepatoprotective Properties of a Polyphenol-Enriched Fraction from *Annona crassiflora* Mart. Fruit Peel against Diabetes-Induced Oxidative and Nitrosative Stress. J. Agric. Food Chem..

[41] Latha M., Pari L. (2004). Effect of an aqueous extract of Scoparia dulcis on blood glucose, plasma insulin and some polyol pathway enzymes in experimental rat diabetes. Braz. J. Med. Biol. Res..

[42] Sheweita S.A., Mashaly S., Newairy A.A., Abdou H.M., Eweda S.M. (2016). Changes in Oxidative Stress and Antioxidant Enzyme Activities in Streptozotocin-Induced Diabetes Mellitus in Rats: Role of *Alhagi maurorum* Extracts. Oxidative Med. Cell. Longev..

[43] Syiem D., Warjri P. (2015). Antidiabetic, antioxidant, and TNF-α lowering properties of extract of the traditionally used plant *Ixeris gracilis* in alloxan-induced diabetic mice. Pharm. Biol..

[44] Sharma R.D., Raghuram T.C., Rao N.S. (1990). Effect of fenugreek seeds on blood glucose and serum lipids in type I diabetes. Eur. J. Clin. Nutr..

[45] Gupta A., Gupta R., Lal B. (2001). Effect of *Trigonella foenum-graecum* (fenugreek) seed on glycemic control and insulin re-sistance in type 2 diabetes mellitus: A double blind placebo controlled study. J. Assoc. Physicians. India.

[46] Sharma S., Mishra V., Jayant S.K., Srivastava N. (2015). Effect of *Trigonella foenum graecum* L on the Activities of Antioxidant Enzyme and Their Expression in Tissues of Alloxan-Induced Diabetic Rats. J. Evid.-Based Integr. Med..

[47] Xue W.L., Li X.S., Zhang J., Liu Y.H., Wang Z.L., Zhang R.J. (2007). Effect of *Trigonella foenum-graecium* (fenugreek) extract on blood glucose, blood lipid and haemorhheological properties in streptozotocin induced diabetic rats. Asia. Pac. J. Clin. Nutr..

[48] Mihailović M., Arambašić J., Uskoković A., Dinić S., Grdović N., Marković J., Mujić I., Šijački A., Poznanović G., Vidaković M. (2013). β-Glucan administration to diabetic rats reestablishes redox balance and stimulates cellular prosurvival mechanisms. J. Funct. Foods.

[49] Mihailović M., Arambašić J., Uskoković A., Dinić S., Grdović N., Marković J., Bauder J., Poznanović G., Vidaković M. (2013). beta-Glucan administration to diabetic rats alleviates oxidative stress by lowering hyperglycaemia, decreasing non-enzymatic glycation and protein O-GlcNAcylation. J. Funct. Foods.

[50] Jangale N.M., Devarshi P.P., Dubal A.A., Ghule A.E., Koppikar S.J., Bodhankar S.L., Chougale A.D., Kulkarni M.J., Harsulkar A.M. (2013). Dietary flaxseed oil and fish oil modulates expression of antioxidant and inflammatory genes with alleviation of protein glycation status and inflammation in liver of streptozotocin-nicotinamide induced diabetic rats. Food Chem..

[51] Kassaian N., Azadbakht L., Forghani B., Amini M. (2009). Effect of Fenugreek Seeds on Blood Glucose and Lipid Profiles in Type 2 Diabetic Patients. Int. J. Vitam. Nutr. Res..

[52] Kaatabi H., Bamosa A.O., Badar A., Al-Elq A., Abou-Hozaifa B., Lebda F., Al-Khadra A., Al-Almaie S. (2015). Nigella sativa im-proves glycemic control and ameliorates oxidative stress in patients with type 2 diabetes mellitus: Placebo controlled participant blinded clinical trial. PLoS ONE.

[53] Zemestani M., Rafraf M., Asghari-Jafarabadi M. (2016). Chamomile tea improves glycemic indices and antioxidants status in patients with type 2 diabetes mellitus. Nutrition.

[54] Qian Q., Qian S., Fan P., Huo D., Wang S. (2011). Effect of *Salvia miltiorrhiza* Hydrophilic Extract on Antioxidant Enzymes in Diabetic Patients with Chronic Heart Disease: A Randomized Controlled Trial. Phytotherapy Res..

[55] Crouse J.R., Morgan T., Terry J.G., Ellis J., Vitolins M., Burke G.L. (1999). A Randomized Trial Comparing the Effect of Casein with That of Soy Protein Containing Varying Amounts of Isoflavones on Plasma Concentrations of Lipids and Lipoproteins. Arch. Intern. Med..

[56] Nelson S.K., Bose S.K., Grunwald G.K., Myhill P., McCord J.M. (2006). The induction of human superoxide dismutase and catalase in vivo: A fundamentally new approach to antioxidant therapy. Free Radic. Biol. Med..

[57] De Oliveira B.F., Costa D.C., Nogueira-Machado J.A., Chaves M.M. (2013). β-Carotene, α-tocopherol and ascorbic acid: Differential profile of antioxidant, inflammatory status and regulation of gene expression in human mononuclear cells of diabetic donors. Diabetes Metab. Rev. Res..

[58] Castellano J.M., Guinda A., Delgado T., Rada M., Cayuela J.A. (2013). Biochemical Basis of the Antidiabetic Activity of Oleanolic Acid and Related Pentacyclic Triterpenes. Diabetes.

[59] Vinayagam R., Xu B. (2017). 7, 8-Dihydroxycoumarin (daphnetin) protects INS-1 pancreatic β-cells against streptozotocin-induced apoptosis. Phytomedicine.

[60] Kobori M., Matsumoto S., Akimoto Y., Takahashi Y. (2009). Dietary Quercetin alleviates diabetic symptoms and reduces streptozocin induced disturbance of hepatic gene expression in mice. Mol. Nutr. Food. Res..

[61] Jeong S.M., Kang M.J., Choi H.N., Kim J.H., Kim J.I. (2012). Quercetin ameliorates hyperglycemia and dyslipidemia and improves antioxidant status in type2 diabetic db/db mice. Nutr. Res. Pract..

[62] El-Moselhy M.A., Taye A., Shaekawi S.S., El-Sisi S.F., Ahmad A.F. (2011). The antihyperglycaemic effects of curcumin on high fat diet fed rats. Role of TNFα and free fatty acids. Food. Chem. Toxicol..

[63] El-Bahr S.M. (2013). Curcumin regulates gene expression of insulin like growth factor, B-cell CLL/lymphoma 2 and antioxidant enzymes in streptozotocin induced diabetic rats. BMC Complement. Altern. Med..

[64] Najafian M., Jahromi M.Z., Nowroznejihad M.J., Khajeaian P., Kargar M.M., Sadeghi M., Arasteh A. (2012). Phlordizin reduces blood glucose levels and improves lipid metabolism in streptozotocin –induced diabetic rats. Mol. Biol. Rep..

[65] Zhang Y., Li X., Zou D., Liu W., Yang J., Zhu N., Huo L., Wang M., Hong J., Wu P. (2008). Treatment of Type 2 Diabetes and Dyslipidemia with the Natural Plant Alkaloid Berberine. J. Clin. Endocrinol. Metab..

[66] Chatuphonprasert W., Lao-Ong T., Jarukamjorn K. (2013). Improvement of superoxide dismutase and catalase in streprozotocin-nicotinamide-induced type 2 diabetes in mice by berberine and glibenclamide. Pharm. Biol..

[67] Kaur K.K., Allahbadia G., Singh M. (2018). Impact of Nutrigenomics on Various Metabolic Disorders in Relation to Life Style Alteration. Austin. J. Nutri. Food. Sci..

[68] Grosso G., Stepaniak U., Micek A., Stefler D., Bobak M., Pająk A. (2017). Dietary polyphenols are inversely associated with metabolic syndrome in Polish adults of the HAPIEE study. Eur. J. Nutr..

[69] Arts I.C., Hollman P.C., Feskens E.J., De Mesquita H.B.B., Kromhout D. (2001). Catechin intake might explain the inverse relation between tea consumption and ischemic heart disease: The Zutphen Elderly Study. Am. J. Clin. Nutr..

[70] Ding Y., Zhang B., Zhou K., Chen M., Wang M., Jia Y., Song Y., Li Y., Wen A. (2014). Dietary ellagic acid improves oxidant-induced endothelial dysfunction and atherosclerosis: Role of Nrf2 activation. Int. J. Cardiol..

[71] Panchal S.K., Poudyal H., Brown L. (2012). Quercetin Ameliorates Cardiovascular, Hepatic, and Metabolic Changes in Diet-Induced Metabolic Syndrome in Rats. J. Nutr..

[72] Qin X., Qiu C., Zhao L. (2014). Maslinic acid protects vascular smooth muscle cells from oxidative stress through Akt/Nrf2/HO-1 pathway. Mol. Cell. Biochem..

[73] Chae Y.J., Kim C.H., Ha T.S., Hescheler J., Ahn H.Y., Sachinidis A. (2007). Epigallocatechin-3-O-gallate inibits the angiotensinII induced adhesion molecule expression in human umbilical vein endothelial cell via inhibition of MAPK pathways. Cell. Physiol. Biochem..

[74] Yang S., Chou G., Li Q. (2018). Cardioprotective role of azafrin in against myocardial injury in rats via activation of the Nrf2-ARE pathway. Phytomedicine.

[75] Yu H., Shi L., Zhao S., Sun Y., Gao Y., Sun Y., Qi G. (2015). Triptolide Attenuates Myocardial Ischemia/Reperfusion Injuries in Rats by Inducing the Activation of Nrf2/HO-1 Defense Pathway. Cardiovasc. Toxicol..

[76] Yu X., Cui L., Zhang Z., Zhao Q., Li S. (2013). -Linolenic acid attenuates doxorubicin-induced cardiotoxicity in rats through suppression of oxidative stress and apoptosis. Acta Biochim. Biophys. Sin..

[77] Szkudelska K., Szkudelski T. (2010). Resveratrol, obesity and diabetes. Eur. J. Pharmacol..

[78] Fang W.-J., Wang C.-J., He Y., Zhou Y.-L., Peng X.-D., Liu S.-K. (2017). Resveratrol alleviates diabetic cardiomyopathy in rats by improving mitochondrial function through PGC-1α deacetylation. Acta Pharmacol. Sin..

[79] Mohammadshahi M., Haidari F., Soufi F.G. (2014). Chronic resveratrol administration improves diabetic cardiomyopathy in part by reducing oxidative stress. Cardiol. J..

[80] Vella R.K., Pullen C., Coulson F., Fenning A.S. (2015). Resveratrol prevents cardiovascular complications in the SHR/STZ rat by reductions in oxidative stress and inflammation. Biol. Med. Res. Int..

[81] Li H., Xia N., Förstermann U. (2012). Cardiovascular effects and molecular targets of resveratrol. Nitric Oxide.

[82] Yang D., Xiao C.X., Su Z.H., Huang M.W., Qin M., Wu W.J., Jia W., Zhu Y., Hu J., Liu X. (2017). (-)-7(S)-hydroxymatairesinol protects against tumor necrosis factor-a-mediated inflammation response in endothelial cells by blocking the MAPK/NF-kB and activating Nrf2/HO-1. Phytomedicine.

[83] Sahu B.D., Kumar J.M., Kuncha M., Borkar R.M., Srinivas R., Sistla R. (2016). Baicalein alleviates doxorubicin-induced cardiotoxicity via suppression of myocardial oxidative stress and apoptosis in mice. Life Sci..

[84] Sarkar P., Nath K., Banu S. (2019). Modulatory effect of baicalein on gene expression and activity of antioxidant enzymes in streptozotocin-nicotinamide induced diabetic rats. Braz. J. Pharm. Sci..

[85] Zeng C., Zhong P., Zhao Y., Kanchana K., Zhang Y., Khan Z.A., Chakrabarti S., Wu L., Wang J., Liang G. (2015). Curcumin protects hearts from FFA-induced injury by activating Nrf2 and inactivating NF-kB both in vitro and in vivo. J. Mol. Cell Cardiol..

[86] Bahadoran Z., Tohidi M., Nazeri P., Mehran M., Azizi F., Mirmiran P. (2012). Effect of broccoli sprouts on insulin resistance in type 2 diabetic patients: A randomized double-blind clinical trial. Int. J. Food Sci. Nutr..

[87] Bai Y., Cui W., Xin Y., Miao X., Barati M.T., Zhang C., Chen Q., Tan Y., Cui T., Zheng Y. (2013). Prevention by sulforaphane of diabetic cardiomyopathy is associated with up-regulation of Nrf2 expression and transcription activation. J. Mol. Cell. Cardiol..

[88] Wu H., Kong L., Cheng Y., Zhang Z., Yangwei W., Manyu L., Yi T., Xiangmei C., Lining M., Lu C. (2015). Metallothionein plays a prominent role in the prevention of diabetic nephropathy by sulforaphane via up-regulation of Nrf2. Free Radic. Biol. Med..

[89] Gu J., Cheng Y., Wu H., Kong L., Wang S., Xu Z., Zhang Z., Tan Y., Keller B.B., Zhou H. (2016). Metallothionein Is Downstream of Nrf2 and Partially Mediates Sulforaphane Prevention of Diabetic Cardiomyopathy. Diabetes.

[90] Zhou J., Wang T., Wang H., Jiang Y., Peng S. (2019). Obacunone attenuates high glucose-induced oxidative damage in NRK-52E cells by inhibiting the activity of GSK-3β. Biochem. Biophys. Res. Commun..

[91] Sadi G., Şahin G., Bostanci A. (2019). Modulation of Renal Insulin Signaling Pathway and Antioxidant Enzymes with Streptozotocin-Induced Diabetes: Effects of Resveratrol. Medicina.

[92] Gao D., Zhao M., Qi X., Liu Y., Li N., Liu Z., Bian Y. (2016). Hypoglycemic effect of *Gynostemma pentaphyllum* saponins by enhancing the Nrf2 signaling pathway in STZ-inducing diabetic rats. Arch. Pharmacal Res..

[93] Mihailović M., Arambašić J., Uskoković A., Dinić S., Grdović N., Marković J., Poznanović G., Vidaković M. (2012). Alpha-lipoic acid preserves the structural and functional integrity of red blood cells by adjusting the redox disturbance and decreasing O-GlcNAc modifications of antioxidant enzymes and heat shock proteins in diabetic rats. Eur. J. Nutr..

[94] Arambašić J., Mihailović M., Uskoković A., Dinić S., Grdović N., Marković J., Poznanović G., Bajec D., Vidaković M. (2013). Alpha-lipoic acid upregulates antioxidant enzyme gene expression and enzymatic activity in diabetic rat kidneys through an O-GlcNAc-dependent mechanism. Eur. J. Nutr..

[95] Ooi B.K., Chan K.-G., Goh B.H., Yap W.H. (2018). The Role of Natural Products in Targeting Cardiovascular Diseases via Nrf2 Pathway: Novel Molecular Mechanisms and Therapeutic Approaches. Front. Pharmacol..

[96] Zhao L., Tao X., Qi Y., Xu L., Yin L., Peng J. (2018). Protective effect of dioscin against doxorubicin-induced cardiotoxicity via adjusting microRNA-140-5p-mediated myocardial oxidative stress. Redox Biol..

[97] Mattagajasingh I., Kim C.-S., Naqvi A., Yamamori T., Hoffman T.A., Jung S.-B., DeRicco J., Kasuno K., Irani K. (2007). SIRT1 promotes endothelium-dependent vascular relaxation by activating endothelial nitric oxide synthase. Proc. Natl. Acad. Sci. USA.

[98] Tomé-Carneiro J., Larrosa M., Yáñez-Gascón M.J., Dávalos A., Gil-Zamorano J., Gonzálvez M., García-Almagro F.J., Ros J.A.R., Tomás-Barberán F.A., Espín J.C. (2013). One-year supplementation with a grape extract containing resveratrol mod-ulates inflammatory-related microRNAs and cytokines expression in peripheral blood mononuclear cells of type 2 diabetes and hypertensive patients with coronary artery disease. Pharmacol. Res..

[99] Ekor M. (2014). The growing use of herbal medicines: Issues relating to adverse reactions and challenges in monitoring safety. Front. Pharmacol..

[100] Epriliati I., Ginjom R. (2012). Bioavailability of Phytochemicals, Phytochemicals—A Global Perspective of Their Role in Nutrition and Health.

[101] Sun S., Wang Y., Wu A., Ding Z., Liu X. (2019). Influence factors of the pharmacokinetics of herbal resourced compounds in clinical practice. Evid.-Based Complem. Alt. Med..

[102] Gambini J., Inglés M., Olaso G., Lopez-Grueso R., Bonet-Costa V., Gimeno-Mallench L., Mas-Bargues C., Abdelaziz K.M., Gomez-Cabrera M.C., Vina J. (2015). Properties of Resveratrol: In Vitro and In Vivo Studies about Metabolism, Bioavailability, and Biological Effects in Animal Models and Humans. Oxid. Med. Cell. Longev..

[103] Rein M.J., Renouf M., Cruz-Hernandez C., Actis-Goretta L., Thakkar S.K., Da Silva Pinto M. (2013). Bioavailability of bioactive food compounds: A challenging journey to bioefficacy. Br. J. Clin. Pharmacol..

[104] Zhao Q., Luan X., Zheng M., Tian X.-H., Zhao J., Zhang W.-D., Ma B.-L. (2020). Synergistic Mechanisms of Constituents in Herbal Extracts during Intestinal Absorption: Focus on Natural Occurring Nanoparticles. Pharmaceutics.

[105] Rotter S., Beronius A., Boobis A.R., Hanberg A., van Klaveren J., Luijten M., Machera K., Nikolopoulou D., van der Voet H., Zilliacus J. (2018). Overview on legislation and scientific approaches for risk assessment of combined expo-sure to multiple chemicals: The potential EuroMix contribution. Crit. Rev. Toxicol..

[106] Ifeoma O., Oluwakanyinsola S., Gowder S.J.T. (2013). Screening of herbal medicines for potential toxicities. New Insight in Toxicity and Drug Testing.

